# TDP2–Dependent Non-Homologous End-Joining Protects against Topoisomerase II–Induced DNA Breaks and Genome Instability in Cells and *In Vivo*

**DOI:** 10.1371/journal.pgen.1003226

**Published:** 2013-03-07

**Authors:** Fernando Gómez-Herreros, Rocío Romero-Granados, Zhihong Zeng, Alejandro Álvarez-Quilón, Cristina Quintero, Limei Ju, Lieve Umans, Liesbeth Vermeire, Danny Huylebroeck, Keith W. Caldecott, Felipe Cortés-Ledesma

**Affiliations:** 1 Genome Damage and Stability Centre, University of Sussex, Falmer, United Kingdom; 2 Centro Andaluz de Biología Molecular y Medicina Regenerativa (CABIMER), CSIC–Universidad de Sevilla (Departamento de Genética), Sevilla, Spain; 3 Laboratory of Molecular Biology (Celgen), Department of Development and Regeneration, University of Leuven, Leuven, Belgium; University of Washington, United States of America

## Abstract

Anticancer topoisomerase “poisons” exploit the break-and-rejoining mechanism of topoisomerase II (TOP2) to generate TOP2-linked DNA double-strand breaks (DSBs). This characteristic underlies the clinical efficacy of TOP2 poisons, but is also implicated in chromosomal translocations and genome instability associated with secondary, treatment-related, haematological malignancy. Despite this relevance for cancer therapy, the mechanistic aspects governing repair of TOP2-induced DSBs and the physiological consequences that absent or aberrant repair can have are still poorly understood. To address these deficits, we employed cells and mice lacking tyrosyl DNA phosphodiesterase 2 (TDP2), an enzyme that hydrolyses 5′-phosphotyrosyl bonds at TOP2-associated DSBs, and studied their response to TOP2 poisons. Our results demonstrate that TDP2 functions in non-homologous end-joining (NHEJ) and liberates DSB termini that are competent for ligation. Moreover, we show that the absence of TDP2 in cells impairs not only the capacity to repair TOP2-induced DSBs but also the accuracy of the process, thus compromising genome integrity. Most importantly, we find this TDP2-dependent NHEJ mechanism to be physiologically relevant, as *Tdp2*-deleted mice are sensitive to TOP2-induced damage, displaying marked lymphoid toxicity, severe intestinal damage, and increased genome instability in the bone marrow. Collectively, our data reveal TDP2-mediated error-free NHEJ as an efficient and accurate mechanism to repair TOP2-induced DSBs. Given the widespread use of TOP2 poisons in cancer chemotherapy, this raises the possibility of TDP2 being an important etiological factor in the response of tumours to this type of agent and in the development of treatment-related malignancy.

## Author Summary

DNA double-strand breaks (DSBs) are dangerous because they can lead to cellular death and tissue degeneration if not repaired, or to genome rearrangements, which are a common hallmark of cancer, if repaired incorrectly. Although required for all chromosomal transitions in cells, transient DNA cleavage by topoisomerase II (TOP2) is a potential endogenous source of DSBs, which are characteristic in that TOP2 remains covalently bound to the DNA termini. In addition, numerous chemotherapeutic regimes rely on compounds that “poison” TOP2 activity, stimulating the formation of DSBs that target tumour cells. However, these compounds also affect healthy tissue and confer undesirable side effects, including the stimulation of genome rearrangements that can trigger secondary malignancies (mainly acute leukemia). Identifying the factors that participate in the repair of TOP2-induced DSBs and fully understanding their mechanism of action are therefore important for the design of chemotherapeutic regimes that are more effective and safer. Here we demonstrate that TDP2, a recently identified protein that can liberate DSB termini from blocked TOP2, functions as part of established cellular DSB repair processes and is required to safeguard genome integrity upon treatment with TOP2 poisons, both in cells and in mice. These results can therefore have important implications in cancer treatment.

## Introduction

The double-stranded helical structure of DNA creates topological problems in all processes that involve opening of the double helix and accessing the genetic information [1], [2]. In particular, the transcription and duplication of DNA and its condensation into chromosomes generates knots and tangles that need to be resolved to avoid interference with diverse cellular processes and to ensure faithful chromosome segregation during mitosis. DNA topoisomerases are enzymes that introduce transient breaks in DNA to solve these topological problems. Type II topoisomerases, such as topoisomerase II in eukaryotes (TOP2) are essential homodimeric enzymes that relax, unknot and decatenate DNA molecules by catalyzing the passage of duplex DNA through a transient DNA double strand break (DSB) created by the enzyme [3]. Two isoforms of TOP2, α and β, exist in higher eukaryotes, with primary roles in replication and chromosome segregation and in transcription, respectively.

A key intermediate of TOP2 activity is the cleavage complex, in which each of two topoisomerase subunits is covalently linked to the 5′-terminus of an enzyme-generated DSB via a phosphodiester bond between the active-site tyrosine and the 5′-phosphate. The cleavage complex is normally a very short-lived intermediate, because the topoisomerase rapidly re-ligates the DSB once DNA strand passage through the DSB has occurred. However, under certain circumstances, such as the presence of nearby DNA lesions, cleavage complexes can be stabilized resulting in an increased likelihood of collision with RNA or DNA polymerases [4]. Such collisions can convert cleavage complexes into potentially clastogenic or lethal DSBs that require cellular DNA repair pathways for their removal.

Cleavage complexes are the target of a widely used class of anti-tumor agents that ‘poison’ topoisomerase activity, thereby prolonging the half-life of the intermediate and increasing the possibility of DSB formation [4], [5]. Thus, these drugs kill tumor cells by inducing high levels of TOP2-associated DSBs. Consequently, TOP2 poisons are commonly used antineoplastic drugs in the treatment of a broad range of tumor types including malignant lymphomas, sarcomas, leukemias, and lung, ovarian, breast and testicular cancers [5]. However, similar to other chemotherapeutic agents, TOP2-targeting drugs are only partially selective for tumour cells, resulting in unwanted toxicity in normal tissues and in therapy-associated chromosome translocations and secondary leukemias [6]–[14]. Moreover, some breakpoints in such translocations have actually been correlated with preferential sites of cleavage by TOP2 [13]–[17].

A characteristic feature of TOP2-induced DNA breaks is covalent attachment of the enzyme to 5′ ends of the DNA, which must be removed by cellular end-processing enzymes if DSB repair is to occur [18]. Until recently, the only known mechanism for removal TOP2 peptide from DNA 5′-termini in mammalian cells involved excision of the DNA fragment linked to the peptide using nucleases such as the MRN complex, CtIP or Artemis [19]–[21]. Recently, however, we identified a human 5′-tyrosyl DNA phosphodiesterase (5′-TDP) that can cleave 5′-phosphotyrosyl bonds and thereby release TOP2 from DSB termini without the need to also remove DNA sequence [22]. Consequently, this enzyme, which was previously known as signalling protein and transcription cofactor TTRAP/EAPII [23], [24], is now denoted tyrosyl DNA phopshodiesterase-2 (TDP2; Human Gene Nomeclature Organisation). Notably, consistent with its enzyme activity, TDP2 is required for cellular resistance to the anti-cancer TOP2 poison etoposide, but is not required for cellular resistance to ionizing radiation or methylmethane sulphonate [22], [25]; agents that induce DNA damage independently of TOP2 activity.

Following DNA end processing, DSBs can be repaired either by homologous recombination (HR) or by non-homologous end joining (NHEJ) [26]. However, these pathways utilize fundamentally different mechanisms for rejoining DSBs and consequently differ in their accuracy. In particular, HR utilizes undamaged sister chromatids to replace any nucleotides removed from DNA termini during DNA end processing and consequently is normally ‘error-free’. However, this process is available only during S phase or G2, when sister chromatids are available. In contrast, NHEJ is a ‘cut-and-splice’ process in which DSB termini are ligated together following DNA end processing without accurate replacement of missing nucleotides, and thus is potentially ‘error-prone’.

Here, we employ avian and murine experimental models to show that *TDP2/Tdp2* deletion results in hypersensitivity to a structurally diverse range of anti-cancer TOP2 poisons. Moreover, we present genetic, biochemical and cellular evidence for TDP2 functioning in a mechanism of NHEJ that protects genome integrity in response to TOP2-induced damage. Finally, we show that this TDP2 dependent pathway also operates *in vivo*, as, upon exposure to TOP2 poisons, it is required for normal adult mouse lymphopoiesis, intestinal mucosa homeostasis and the maintenance of genome stability in the bone marrow. Collectively, our results suggest that TDP2 defines an error-free mechanism of NHEJ in mammals, which is specialized in the repair of TOP2-induced DSBs and reduces both tissue toxicity and genome instability in response to this particular type of DNA damage. These findings suggest the possibility of TDP2 being a significant etiological factor in the clinical tolerance and response to widely used TOP2 poisons.

## Results

### TDP2 is required for cellular resistance to clinical TOP2 poisons and is the major 5′-TDP activity in the mouse

**Figure 1:**
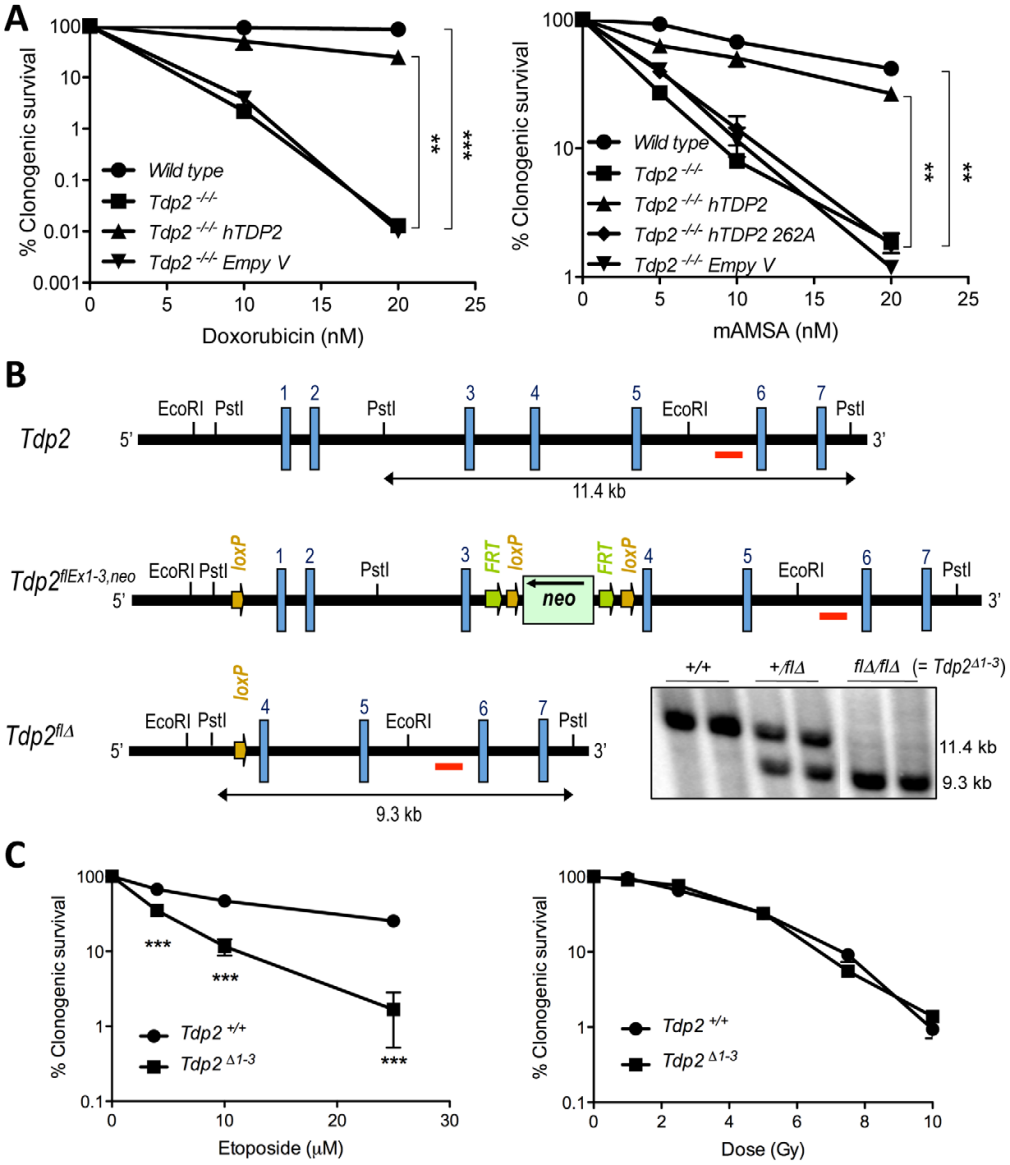
TDP2 promotes survival following TOP2-induced DSBs. A. Clonogenic survival of the indicated DT40 cell line; wild-type, *TDP2^−/−/−^* and *TDP2^−/−/−^* complemented with human TDP2 (hTDP2) and catalytic-dead human TDP2 (hTDP2 262A) or empty vector (Empty V); following continuous treatment with the indicated concentrations of doxorubicin (left) or mAMSA (right). Average ± s.e.m. of at least three independent experiments and statistical significance at the highest indicated dose when compared to *TDP2^−/−/−^* cells by Two-way ANOVA with Bonferroni post-test is shown. B. Scheme showing the strategy for targeted deletion of the first three exons of *Tdp2* in mouse. The wild-type (*Tdp2^+^*), conditional (*Tdp2^flEx1–3,neo^*) and deleted (*Tdp2^flΔ^*) alleles are depicted. The *EcoRI-EcoRI* fragment of *Tdp2* was used in the targeting construct. Southern-blot analysis of *PstI*-digested DNA from wild-type (*+/+*), heterozygous (*+/flΔ*) and knock-out (*flΔ/flΔ*, from now on denoted *Tdp2^Δ1–3^*) mice, using the indicated probe (red line), is shown (bottom right). C. Clonogenic survival of wild-type and *Tdp2^Δ1–3^* transformed MEFs after 3 h acute exposure to the indicated concentrations of etoposide (left) or the indicated dose of γ-irradiation (right). Average ± s.e.m. of three independent experiments and statistical significance by Two-way ANOVA test with Bonferroni post-test is shown. In all figures (*P≤0.05; **P≤0.01; ***P≤0.005).

The discovery of TDP2 as the first 5′-TDP activity raised the possibility of it being an important factor in the clinical response to TOP2 poisons [22], [25]. Indeed, TDP2 deleted avian DT40 cells are hypersensitive to etoposide [22], [25]. To address this question further, we examined the sensitivity of *TDP2^−/−/−^* cells to two additional, structurally diverse, TOP2 poisons. These drugs, denoted doxorubicin and amsacrine (m-AMSA), are employed widely during cancer therapy but in contrast to etoposide, ‘poison’ TOP2 by intercalating into DNA [5]. Nevertheless, similarly to etoposide, *TDP2^−/−/−^* cells displayed significant hypersensitivity to both doxorubicin and m-AMSA (Figure 1A). Moreover, a functional TDP2 phosphodiesterase domain was required for cellular resistance to this type of drug, because expression of wild-type human TDP2 (hTDP2) rescued the sensitivity of *TDP2^−/−/^* ^−^ DT40 cells to m-AMSA, whereas hTDP2^D262A^ harbouring an inactivating mutation in the catalytic active site [5] did not (Figure 1A). These results show that TDP2 is required for cellular resistance to a range clinically relevant and structurally diverse TOP2 poisons, and support our contention that this requirement reflects the 5′-TDP activity of this enzyme.

To determine the impact of TDP2 on TOP2-induced DNA damage in mammals, and thus its possible relevance to anti-cancer therapy, we adopted a mouse model in which the first three exons of *Tdp2*, plus the 5′-UTR, were deleted by Cre-mediated excision (Figure 1B; see Materials and Methods). Mice homozygous for the deleted allele (*Tdp2^flΔ^*, from here-on denoted *Tdp2^Δ1–3^*) are viable, and so far we have not detected any abnormal pathology (unpublished observations). However, transformed *Tdp2^Δ1–3^* mouse embryonic fibloblasts (MEFs) were hypersensitive to etoposide (Figure 1C, left, and Figure S1), but were not hypersensitive to DNA damage induced independently of TOP2 by γ-irradiation (Figure 1C, right).

**Figure 2:**
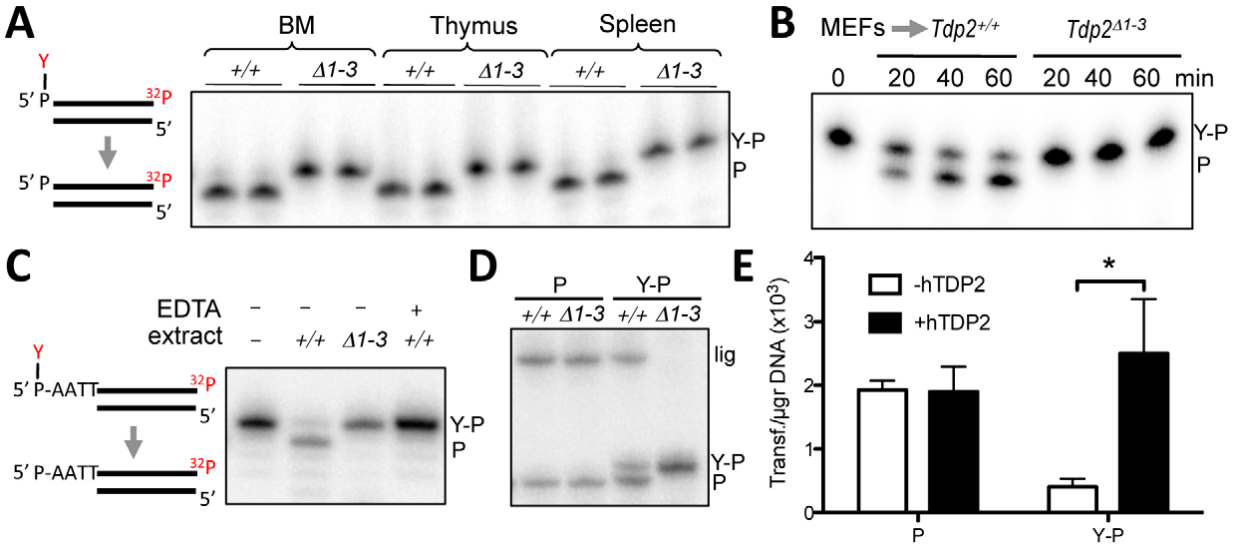
Deletion of *Tdp2* in mouse abolishes 5′-TDP activity and ligation of 5′ phosphotyrosine-blocked ends. A. Duplex substrate harbouring a 5′phosphotyrosine blunt end (left) was incubated with 9 µg *Tdp2^+/+^* or *Tdp2^Δ1–3^* tissue extract from bone marrow (BM), thymus and spleen for 1 h. B. Substrate in “A” was incubated with 1.5 µg of cellular extract from *Tdp2^+/+^* or *Tdp2^Δ1–3^* primary MEFs for the indicated time. C. Duplex substrate harbouring a 5′phosphotyrosine self-complementary overhang end (left) was incubated with 10 µg cellular extract from *Tdp2^+/+^* or *Tdp2^Δ1–3^* transformed MEFs for 2 h in the presence or absence of 50 mM EDTA. D. Self-ligation of 5′ phosphate (P) and 5′ phosphotyrosine (Y–P) overhang substrates as depicted in “C” incubated for 1.5 h with 3.3 µg cellular extract from *Tdp2^+/+^* or *Tdp2^Δ1–3^* transformed MEFs in the presence of T4 DNA ligase. In all cases migration of the 5′ phosphotyrosine substrate (Y–P), 5′ phosphate (P) and ligation (lig) products are indicated. E. Circularization efficiency of a linear plasmid with 5′ phosphotyrosine (Y–P) and 5′ phosphate (P) catalysed by *Tdp2^Δ1–3^* transformed MEFs extracts in the presence and absence of recombinant human TDP2 (hTDP2). Reaction products were transformed into *E. coli* and the number of transformants obtained per µg of initial substrate DNA (average ± s.e.m. of three independent experiments) is shown. Statistical significance by Two-way ANOVA test with Bonferroni post-test is indicated is shown.

Protein extracts from spleen, thymus, and bone marrow from wild type mice possess robust 5′-TDP activity, but, importantly, this activity was absent in analogous protein extracts from *Tdp2^Δ1–3^* mice, confirming successful inactivation of the enzyme (Figure 2A). Cell extracts prepared from primary *Tdp2^Δ1–3^* MEFs also lacked detectable 5′-TDP activity (Figure 2B). This was true not only for blunt-ended DSB substrates, but also for DSB substrates harbouring a 4-bp 5′-overhang (Figure 2C), characteristic of TOP2-induced DSBs. Additionally, EDTA-mediated chelation of Mg^2+^, which is essential for TDP2 function, completely eliminates 5′-TDP activity in wild type MEF extracts. These observations are significant because the related enzyme TDP1, whose activity is Mg^2+^ independent, was recently reported to possess weak activity on this type of substrate [27]. Our data therefore suggest that TDP2 is the primary, if not only, source of 5′-TDP activity in MEF extracts (Figure 2C).

### TDP2 creates ligatable DSBs and functions in NHEJ

Based on the mechanism of TOP2 cleavage, we anticipated that TDP2 activity would reconstitute ‘clean’ DSBs (5′ phosphate and 3′ hydroxyl termini) with 4-bp overhangs, which would be an ideal substrate for ligation by NHEJ. Interestingly, these ligation events would accurately preserve the DNA sequence, suggesting the possibility of an error-free NHEJ mechanism that specifically acts on TOP2-induced DSBs. To test this hypothesis, we examined whether TDP2 action at DSBs typical of those induced by TOP2 creates termini that can be ligated by T4 DNA ligase. Indeed, inclusion of T4 DNA ligase in reactions containing wild type MEF extract resulted in the additional appearance of a product of 46-nt, indicative of the completion of DSB repair by DNA ligation. However, this product was not observed if reactions contained cell extract from *Tdp2^Δ1–3^* MEFs, confirming that DNA ligation was dependent on TDP2 activity (Figure 2D). Interestingly, the length of the product is consistent with a ligation event in which DNA sequence is preserved. To analyse ligation events directly catalysed by cell extracts, we generated linear plasmids harbouring 5′ phosphate or 5′ phosphotyrosine ends by PCR amplification with the corresponding modified primers. The incubation of these substrates with NHEJ-competent nuclear extracts [28] results in plasmid circularization events that can be scored as colonies following bacterial transformation. As can be seen in Figure 2E, nuclear extracts from *Tdp2^Δ1–3^* MEFs efficiently circularized linear plasmids with 5′ phosphate ends but not linear plasmids harbouring 5′-phosphotyrosine. This difference was lost upon addition of recombinant TDP2 to the reaction, confirming the TDP2–dependent nature of the repair reaction. Collectively, our data suggest that TDP2 activity facilitates NHEJ of 5′ tyrosine-blocked ends by generating DSBs with ligatable termini, consistent with our hypothesis that this enzyme can support error-free NHEJ of TOP2-induced DNA damage.

**Figure 3:**
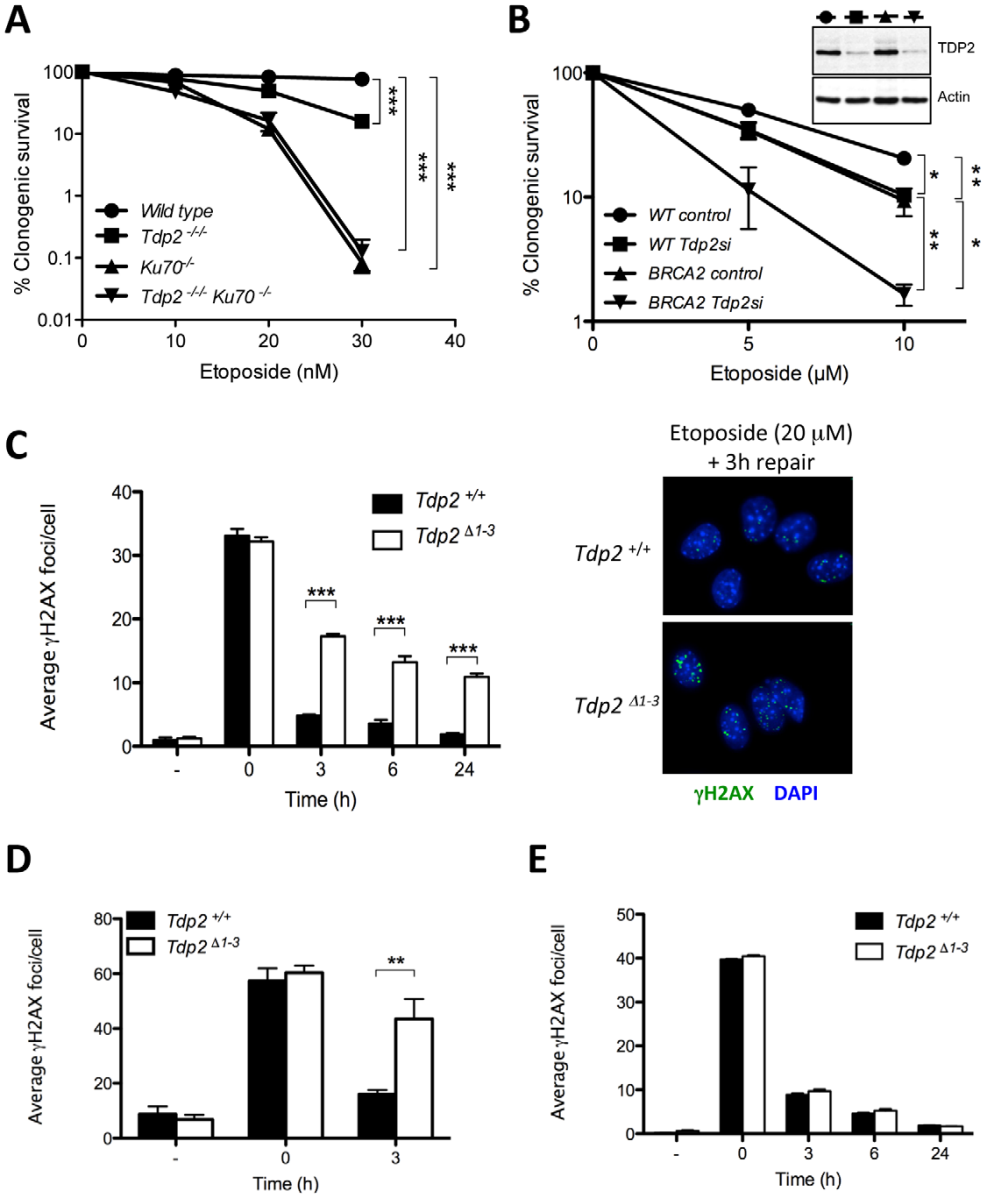
TDP2 promotes repair of TOP2-induced DSBs by NHEJ. A. Clonogenic survival of wild-type, *TDP2^−/−/−^*, *KU70^−/−^* and *TDP2^−/−/−^ KU70^−/−^* DT40 cells following continuous treatment with the indicated concentrations of etoposide. Average ± s.e.m. of at least three independent experiments and statistical significance at the highest indicated dose by Two-way ANOVA with Bonferroni post-test is shown. B. Clonogenic survival of wild-type and BRCA2-mutant human transformed fibroblasts with (Tdp2si) and without (control) TDP2 depletion following 3 h acute exposure to the indicated concentrations of etoposide. Western blot analysis of TDP2 levels in wild type and BRCA2-mutant cell extracts after 48 h of transfection is indicated (inset). Other details as in “A”. C. γH2AX foci induction after 30 min 20 µM etoposide treatment and repair at different times following drug removal in confluency arrested *Tdp2^+/+^* and *Tdp2^Δ1–3^* primary MEFs. Representative images of the 3 h repair time point including DAPI counterstain (right) and average ± s.e.m. of at least three independent experiments (left) are shown. Statistical significance by Two-way ANOVA test with Bonferroni post-test is indicated. D. G2 primary MEFs (see Matherials and Methods) following 30 min 10 µM etoposide treatment. Other details as in “C”. E. Confluency arrested primary MEFs exposed to 2Gy γ-irradiation. Other details as in “C”.

To genetically test whether TDP2 functions indeed during NHEJ, we generated *TDP2^−/−/−^* DT40 cells harboring a targeted deletion of Ku70, a core component of the NHEJ pathway (Figure S2). Whilst both *TDP2^−/−/−^* and *KU70^−/−^* cells were hypersensitive to etoposide, cells in which both genes were deleted (*TDP2^−/−/−^*/*KU70^−/−^*) were no more hypersensitive than cells in which Ku70 alone was deleted (Figure 3A). In contrast to this epistatic relationship with a core NHEJ factor, transient knockdown of TDP2 further enhances etoposide sensitivity of HR defective (BRCA2 mutated) human fibroblasts (Figure 3B). Based on these genetic relationships, we conclude that TDP2 functions in a NHEJ-mediated and HR-independent pathway for the repair of TOP2-induced DSBs.

To further assign a role for TDP2 in the NHEJ pathway for DSB repair, we measured DSB repair rates in primary *Tdp2^Δ1–3^* MEFs by immunodetection of γH2AX, a phosphorylated derivative of histone H2AX that arises at sites of chromosomal DSBs [29]. We measured DSB repair in specific phases of the cell cycle, because whilst NHEJ is operative throughout, HR-mediated DSB repair is operative only in S/G2 [30]. Notably, DSB repair rates were markedly reduced in *Tdp2^Δ1–3^* MEFs following etoposide treatment, both in G0/G1 (Figure 3C) and G2 (Figure 3D), consistent with TDP2 functioning, as NHEJ, independently of cell cycle. These results were not specific to murine cells, since similar results were observed in TDP2-depleted human A549 cells (Figure S4). In contrast to treatment with etoposide, the rate of DSB repair was normal in *Tdp2^Δ1–3^* MEFs following γ-irradiation, consistent with a role for TDP2 specifically at TOP2-induced DSBs (Figure 3E). Collectively, these data demonstrate that TDP2 is required in mammalian cells for rapid repair of TOP2-induced DSBs by NHEJ, and for cellular resistance to these lesions.

### TDP2 promotes genome stability following TOP2-induced DNA damage

**Figure 4:**
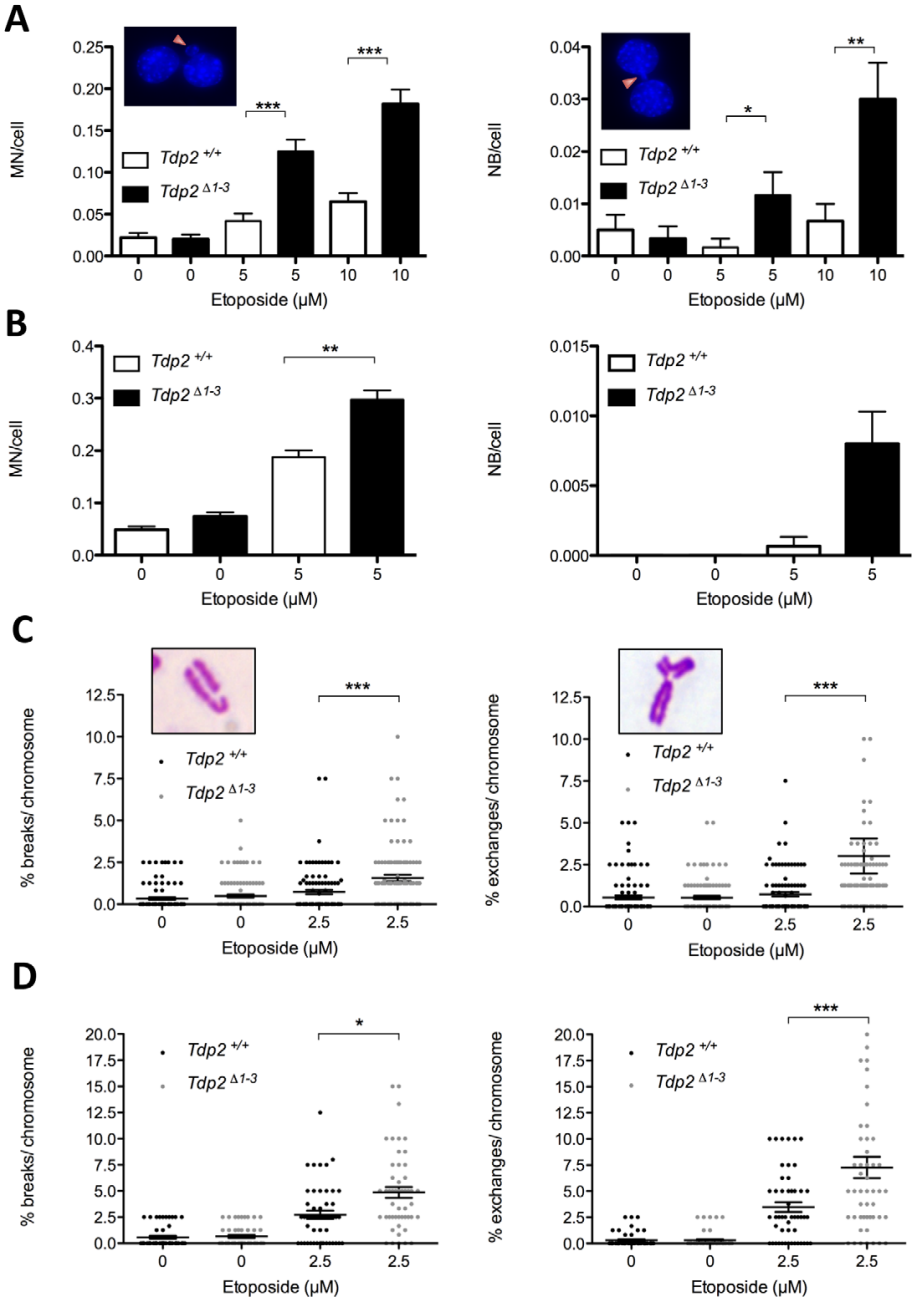
The absence of TDP2 increases etoposide-induced genome instability in mammalian cells. A. Micronuclei (MN, left) and nucleoplasmic bridges (NB, right) in binucleated (following cytochalasin B-mediated cell cycle arrest) *Tdp2^+/+^* and *Tdp2^Δ1–3^* transformed MEFs following acute treatment (30 min) with indicated dose of etoposide. See insets for representative images. Histogram bars represent the average ± s.e.m. of n≥600 cells coming from three independent experiments. Statistical significance by Mann-Whitney test. B. Primary MEFs in the absence of cytochalasin B treatment (n≥1500). Other details as in “A”. C. Break-type (left) and exchange-type (right) chromosomal aberrations in transformed *Tdp2^+/+^* and *Tdp2^Δ1–3^* MEFs following acute treatment (30 min) with indicated dose of etoposide. See insets for a representative image. Plots show the number of breaks/exchanges per 100 chromosomes from individual metaphase spreads (n = 100) obtained in at least two independent experiments. Average ± s.e.m. and statistical significance by Mann-Whitney test is also indicated. D. Metaphase spreads from primary MEFs (n = 50). Caffeine was added 4 h after etoposide treatment. Other details as in “C”.

We hypothesized that this TDP2-mediated error-free NHEJ mechanism would be important to maintain genome integrity upon exposure to TOP2 poisons. To address this possibility, we quantified the frequency of micronuclei (MN), nucleoplasmic bridges (NB), and chromosomal aberrations following etoposide treatment. These events constitute well-established indicators of genome instability caused by misrepair of DSBs in which acentric, dicentric and aberrant chromosomes or chromosome fragments can be formed. As expected, etoposide increased the number of micronuclei and nucleoplasmic bridges in both transformed *Tdp2^+/+^* and *Tdp2^Δ1–3^* MEFs, but this increase was significantly higher (up to three-fold) in *Tdp2^Δ1–3^* cells (Figure 4A). Primary *Tdp2^Δ1–3^* MEFs at low passage (P3–4) similarly displayed elevated levels of micronuclei and nucleoplasmic bridges following etoposide treatment, compared to wild type primary MEFs (Figure 4B), although in the case of nucleoplasmic bridges the low number of cells displaying these structures prevented the difference from reaching statistical significance.

An additional indicator of genome instability is elevated frequencies of chromosome aberrations. Consequently, we quantified the frequency of chromosome breaks and exchanges in metaphase spreads of transformed *Tdp2^+/+^* and *Tdp2^Δ1–3^* MEFs. In agreement with the increased cell cycle arrest of *TDP2^−/−/−^* DT40 cells in G2 following etoposide treatment [25], we noted an etoposide-dependent reduction in metaphase cells that was particularly severe in *Tdp2^Δ1–3^* MEFs (unpublished observations). However, of those metaphases observed and scored, both chromosome exchanges and breaks were significantly higher (2 to 5-fold) in *Tdp2^Δ1–3^* MEFs than in *Tdp2^+/+^* MEFs (Figure 4C). A similar increase in these events in *Tdp2^Δ1–3^* MEFs, compared to wild type cells, was observed if low-passage primary MEFs were employed, ruling out the possibility that the elevated genome instability in *Tdp2^Δ1–3^* MEFs was an artefact of cellular transformation (Figure 4D). In the latter case, etoposide treatment almost ablated the appearance of mitotic cells in populations of both wild type and *Tdp2^Δ1–3^* MEFs, necessitating the use of caffeine to prevent G2 arrest. Taken together these results demonstrate that loss of TDP2 results in increased genome instability following TOP2-induced DNA strand breakage.

### Loss of TDP2 results in elevated homologous recombination

**Figure 5:**
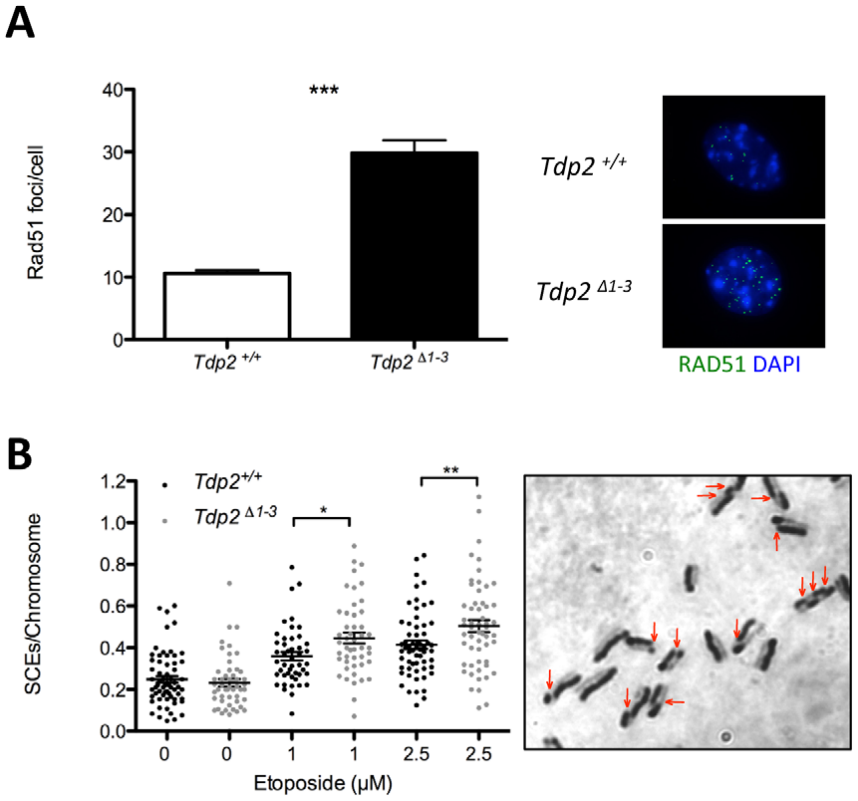
The absence of TDP2 increases etoposide induced homologous recombination. A. Total number of foci per RAD51 foci-containing cell in *Tdp2^+/+^* and *Tdp2^Δ1–3^* primary MEFs following 30 min 10 µM etoposide treatment and 2 h recovery (left). Replicating cells were excluded from the analysis. A representative image is shown (right). Average ± s.e.m. from 3 independent experiments and statistical significance by T test is indicated. B. Sister chromatid exchanges (SCEs) scored in *Tdp2^+/+^* and *Tdp2^Δ1–3^* transformed MEFs after 30 min acute treatment with the indicated concentration of etoposide. Plots show the number of SCEs per chromosome from individual metaphase spreads (n≥50) obtained in at least two independent experiments. Average ± s.e.m. and statistical significance by Mann-Whitney test is also indicated.

The above results demonstrate increased genome instability in *Tdp2^Δ1–3^* MEFs, consistent with a role for TDP2 in error-free NHEJ-mediated repair of TOP2-induced DSBs. In this scenario, we considered the possibility that loss of TDP2 might also result in channelling of DSB repair towards HR. To address this question, we analyzed the formation of RAD51 foci, a well-established indicator of repair by HR. Following treatment with etoposide, the average number of Rad51 foci per cell was ∼3-fold higher in *Tdp2^Δ1–3^* than in wild-type MEFs (Figure 5A), in agreement with an increase in the use of HR to repair TOP2-induced DSBs when TDP2 is not present. Furthermore, we compared the frequency of etoposide-induced sister chromatid exchanges (SCEs), a molecular hallmark of HR [31], in wild type and *Tdp2^Δ1–3^* MEFs (Figure 5B). Notably, SCE levels increased substantially in transformed MEFs following acute etoposide exposure, being significantly higher in *Tdp2^Δ1–3^* cells at two etoposide concentrations tested (1 and 2.5 µM). These data confirm that, upon etoposide treatment, the frequency of HR is elevated in *Tdp2^Δ1–3^* MEFs, consistent with TDP2 functioning in NHEJ.

### Elevated hypersensitivity to TOP2-induced DNA damage in *Tdp2^Δ1–3^* mice

**Figure 6:**
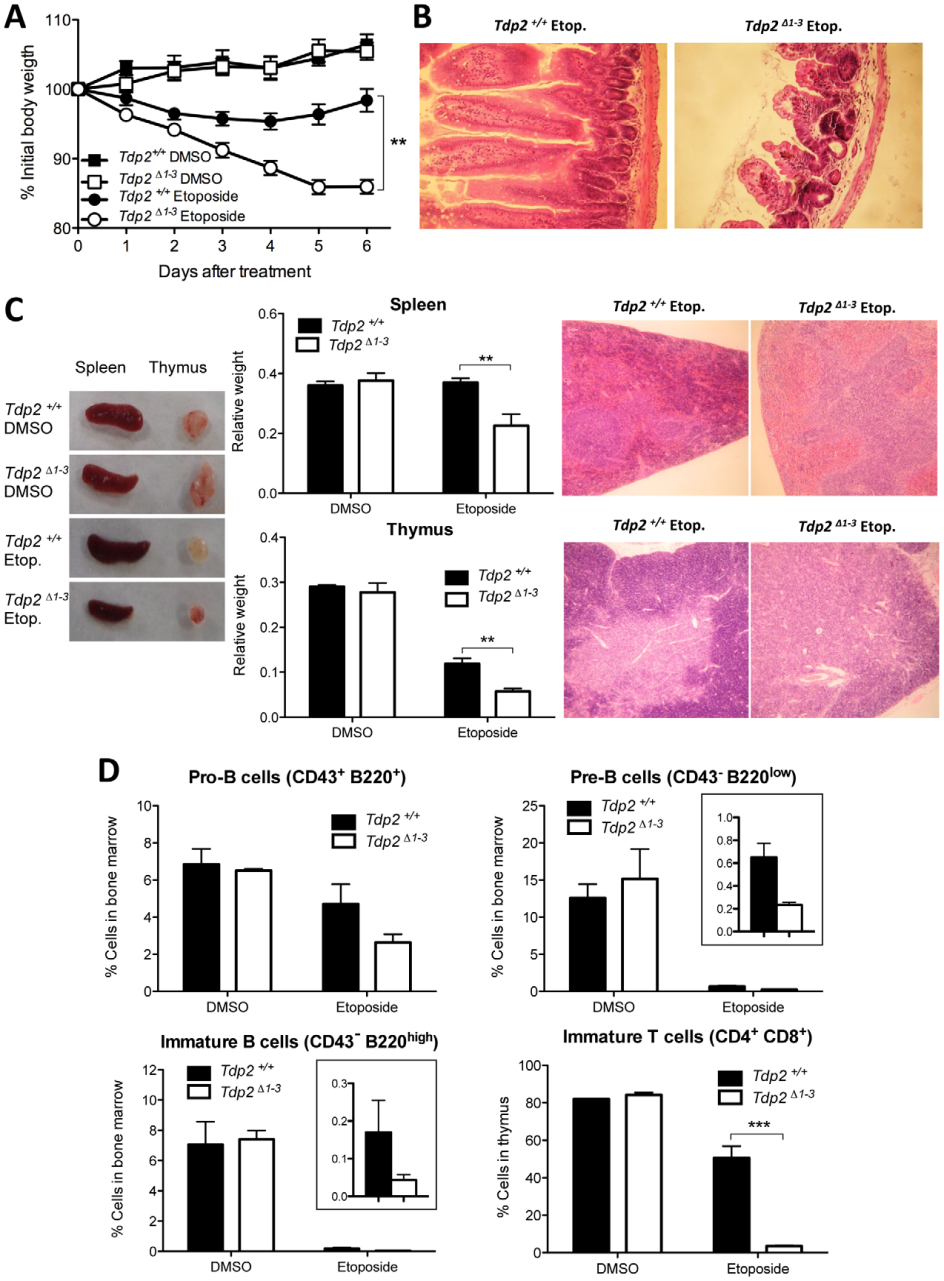
The absence of TDP2 causes etoposide sensitivity *in vivo*. A. 8-week old wild-type and *Tdp2^Δ1–3^* littermates were intraperitoneally injected with a single 75 mg/kg dose of etoposide or vehicle (DMSO) and body weight was recorded in the following 6 days. Average ± s.e.m. of the percentage of initial body weight from at least 8 mice and statistical significance by One-way ANOVA with Bonferroni post-test is shown. B. Representative image of hematoxylin-eosin stained jejunum slices from wild-type and *Tdp2^Δ1–3^* animals 6 days after etoposide treatment. C. Macroscopic (left) and histological (right) representative image of spleen and thymus from wild-type and *Tdp2^Δ1–3^* animals 6 days after treatment. Average weight of these organs ± s.e.m. and statistical significance by Two-way ANOVA with Bonferroni post-test is shown (centre). D. FACS analysis of B-cells in bone marrow (top and bottom-left) and T-cells in thymus (bottom right) in wild-type and *Tdp2^Δ1–3^* animals 6 days after treatment. See insets to compare etoposide treated samples when required. Average percentage of the indicated cell type among the total number of cells in the corresponding tissue ± s.e.m. of at least 3 animals and statistical significance by Two-way ANOVA with Bonferroni post-test is shown.

To address the relevance of TDP2-mediated repair of TOP2-induced DSBs *in vivo*, we compared the impact of etoposide on adult (8 wk) wild type and *Tdp2^Δ1–3^* mice. A single intraperitonal injection of etoposide (75 mg/kg) caused a decrease in body weight in the initial 4 days post-treatment both in wild type and *Tdp2^Δ1–3^* animals (Figure 6A). However, whereas *Tdp2^+/+^* mice exhibited relatively mild and transient weight loss, *Tdp2^Δ1–3^* littermates lost weight progressively and were sacrificed at day 6 to prevent suffering. No differences in body weight were observed between mock-treated (with DMSO) wild type and *Tdp2^Δ1–3^* mice. Histopathological analysis of *Tdp2^Δ1–3^* mice sacrificed 6 days after etoposide treatment revealed marked villous atrophy in the small intestinal mucosa as the likely cause of the drastic weight loss (Figure 6B). This was not observed in either wild-type and/or DMSO treated animals (data not shown), suggesting a protective role for TDP2 against adverse effects of etoposide *in vivo*.

### TOP2-induced DNA damage results in increased lymphoid toxicity in *Tdp2^Δ1–3^* mice

In addition to severe intestinal damage, etoposide administration resulted in elevated splenic and thymic atrophy in *Tdp2^Δ1–3^* mice, compared to wild type mice (Figure 6C), consistent with the known hypersensitivity of these organs to this drug [32]. Histological analysis of these tissues revealed a marked reduction in the cellular content in *Tdp2^Δ1–3^* animals (Figure 6C, right, note the low density of dark-stained nuclei). In light of these results, we analysed B-cell and T-cell maturation in wild type and *Tdp2^Δ1–3^* mice (Figure 6D and Figure S5). In the case of B-cell precursors in bone marrow, treatment with etoposide resulted in a decrease of 30–50% in the fraction of cells that were CD43^+^ B220^+^ progenitors (Pro-B cells) and a decrease of >95% in the fraction of cells that were CD43^−^ B220^low^ (Pre-B cells) or CD43^−^ B220^high^ (immature B cells) precursors. In all cases the reduction in B-cell precursors was greater in *Tdp2^Δ1–3^* mice, but the differences were not statistically significant at the administered dose. In contrast, in the case of T-cell maturation, whereas etoposide treatment reduced the fraction of CD4^+^ CD8^+^ immature T cells by 30–40% in wild type mice, these cells were almost completely eliminated in *Tdp2^Δ1–3^* mice (Figure 6A, bottom right). No effect was observed in CD11b/Mac-1^+^ myeloid cells in the bone marrow (Figure S6). Taken together, these results suggest that loss of TDP2 increases cellular attrition in the lymphoid system, particularly in the T-cell lineage, in response to TOP2-induced DNA damage.

### *Tdp2^Δ1–3^* mice display increased TOP2-induced genome instability in bone marrow

**Figure 7:**
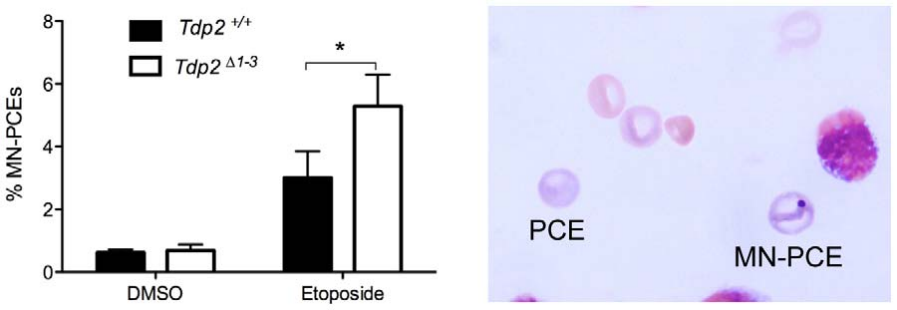
The absence of TDP2 increases etoposide-induced genome instability *in vivo*. Percentage of micronucleated polychromatic erythrocytes (MN-PCE) among the total number of polychromatic erythrocytes (PCE), examples of which are shown (right), in bone marrow smears of wild-type and *Tdp2^Δ1–3^* mice 24 h after intraperitoneal injection of 1 mg/kg etoposide or vehicle (10% DMSO). Average ± s.e.m. of 4 (DMSO) and 6 (VP16) animals and statistical significance by paired T test is shown.

A major side-effect of cancer therapy employing TOP2 poisons is secondary hematological malignancy, and in particular acute leukemia, resulting most likely from error prone/erroneous repair of TOP2-induced DSBs and chromosome translocations [4], [7]. Given our findings that TDP2 limits genome rearrangements induced by etoposide in cells, we examined whether TDP2 also promotes genome stability in bone marrow *in vivo*. We quantified the fraction of micronucleated polychromatic erythrocytes (PCEs) in bone marrow smears from *Tdp2^Δ1–3^* and Tdp2^+/+^ mice 24 hour after intraperitoneal injection of etoposide (1 mg/kg). The rodent erythrocyte micronucleus test is a standard procedure to detect cytogenetic damage in toxicological studies and is based on the detection of micronuclei in erythrocyte precursors (Hayashi et al 1994). As expected, etoposide increased the fraction of PCEs that were micronucleated in both wild type and *Tdp2^Δ1–3^* animals (Figure 7). However, this increase was ∼2-fold higher in *Tdp2^Δ1–3^* mice than in wild type mice, suggesting that TDP2 protects heamatopoietic cells from genome instability induced by anti-cancer TOP2 poisons.

## Discussion

### TDP2 is the major 5′-tyrosyl DNA phosphodiesterase in mammals

In the current study we observe that *Tdp2* deletion ablates detectable 5′-TDP activity in different mouse tissues and MEFs, consistent with our previous observations in DT40 cells [25]. It is worth noting that other roles have been assigned to this protein, in other cellular processes such as signal transduction and transcriptional regulation [33]. So far, however, we have been unable to detect any spontaneous phenotype caused by TDP2 loss, either at cellular level or *in vivo*, while dramatic effects are observed upon etoposide treatment. This suggests that the most important function of TDP2, following Top2 induced DNA damage at least, is related to the 5′ TDP activity of this enzyme. Additionally, our data suggest that alternative, TDP2–independent, mechanisms of DSB repair are sufficient to cope with the endogenous level of TOP2 damage arising during normal mouse development and life.

A role for human TDP1 in repairing TOP2-induced DSBs was recently suggested by a weak 5′-TDP activity of human recombinant protein on DSBs possessing 4-bp 5′-overhangs, and on a mild sensitivity of *TDP1^−/−^* DT40 cells to etoposide [27]. This is also consistent with the increased resistance to etoposide reported in cells highly overexpressing TDP1 [34], and with the reported 5′-TDP activity of Tdp1 in *Saccharomyces cerevisiae* [35]. However, while our standard activity assays employs DSBs with blunt-ended 5′-phosphotyrosyl termini, in the current study we similarly failed to detect residual 5′-TDP activity in *Tdp2^Δ1–3^* MEF extracts on DSB substrates with 4-bp 5′-overhangs (Figure 2C). In addition, in our hands, *TDP1^−/−^* DT40 cells are not hypersensitive to etoposide, and deletion of *TDP1* in *TDP2^−/−/−^* DT40 cells does not increase sensitivity to etoposide above that observed by *TDP2* deletion alone [36]. Consequently, we conclude that TDP2 is the major if not only 5′-TDP activity in mammals (as in DT40 chicken cells), at physiologically relevant enzyme concentrations at least.

### TDP2 is required for survival and efficient repair upon induction of TOP2-mediated DSBs in mammals

We have shown that *Tdp2*-deleted mouse cells are hypersensitive to TOP2-induced DNA damage, but not to ionizing radiation, in agreement with previous results with *TDP2^−/−/−^* DT40 cells [25]. Moreover, we demonstrate that this hypersensitivity correlates with a defect in the repair of etoposide-induced DSBs, as measured by immunostaining for sites of γH2AX, which suggests that TDP2-mediated repair promotes tolerance to TOP2-induced DNA damage in mammalian cells. Remarkably, we observed that TDP2 is required for resistance to TOP2-induced DNA damage not only at the cellular level, but also at the whole-organism level. Indeed, etoposide administration in *Tdp2^Δ1–3^* mice resulted in both increased mortality due to intestinal damage and in elevated toxicity in lymphoid tissue, established *in vivo* targets of etoposide [32]. TDP2 is therefore a critical factor in the cellular and physiological response to TOP2 poisons.

### TDP2 functions in NHEJ and protects genome integrity

One important result of our study was to uncover the relationship between TDP2 and the major DSB-repair pathways, NHEJ and HR. We have shown that TDP2 can convert DSBs with 5′-phosphotyrosyl termini into DSBs that are directly ligatable, and might thus be of particular utility in facilitating an error-free NHEJ pathway for repair of TOP2-induced DSBs. Several of our observations support the idea that TDP2 is a component of NHEJ. First, the contribution of TDP2 to cellular resistance to TOP2 induced DNA damage is dependent on the NHEJ machinery and independent on HR, as, with regards to etoposide sensitivity, *KU70* is epistatic over *TDP2* deletion in DT40 cells while an additive effect is observed when TDP2 is depleted in BRCA2-deficient human fibroblasts. Second, loss of TDP2 results in a DSB repair defect not only in G2 but also in G0/G1, cell cycle stages in which NHEJ is the main if not only DSB repair mechanism available [26], [30], [37], [38]. Third, *Tdp2^Δ1–3^* MEFs exhibit increased levels of HR-mediated DSB repair, as measured by elevated frequencies of RAD51 foci and sister chromatid exchange in response to etoposide treatment, which is a phenotype observed in other cell lines in which NHEJ is defective [39]–[41]. Additionally, we have been unable to generate DT40 cells in which both *TDP2* and *XRCC3* are deleted, suggesting that loss of both TDP2 and HR-mediated DSB repair is cell lethal (unpublished observations).

Whilst the above observations argue strongly that TDP2 is a component of NHEJ, it is important to note that TDP-independent NHEJ mechanisms to process TOP2-linked termini most likely also exist and employ nucleases such as MRN complex, CtIP or Artemis [5], [18]–[21]. This explains why *KU70^−/−^* DT40 cells exhibit much greater hypersensitivity to etoposide than *TDP2^−/−/−^* DT40 cells, and why *Tdp2^Δ1–3^* MEFs still repair a significant fraction of etoposide-induced DSBs in G0/G1 (when NHEJ is the only DSB repair pathway available). Whilst nuclease-mediated NHEJ can support cell survival in response to TOP2-induced DNA damage, they most likely do so at the expense of increased genetic instability. This is because the removal of sequence from 4-bp complementary 5′-overhang during NHEJ will, on the one hand, likely result in chromosome deletions, and on the other hand, increase the propensity for DSB misjoining and chromosome translocation. In contrast, HR provides an error-free pathway to repair TOP2-induced DSBs that have been processed by nucleases, by restoring any missing DNA sequence from and intact sister chromatid in S and G2 [30], [37], [42]. In this scenario, the increased etoposide-induced genome instability in *Tdp2^Δ1–3^* mice, both in cultured cells from these animals and in bone marrow *in vivo*, likely reflects the use of TDP2–independent NHEJ in cellular contexts in which HR-mediated DSB repair is unavailable (e.g. in cells in G0/G1), or is saturated by the number of etoposide-induced DSBs.

**Figure 8:**
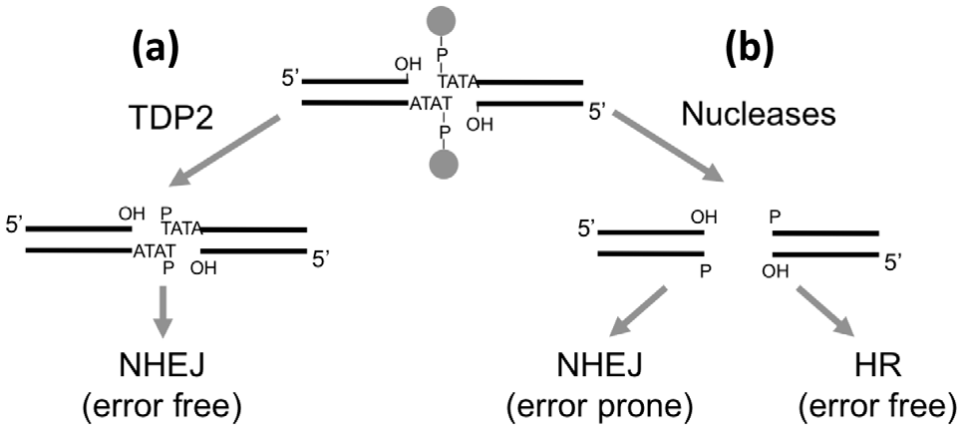
Model for the repair of TOP2-induced DSBs. (a) TDP2-mediated cleavage of the 5′ phosphodiester link between TOP2 peptide and DNA results in 3′ hydroxyl (OH) 5′ phosphste (P) cohesive ends that are easily ligatable by NHEJ resulting in error-free repair. (b) Alternatively, nucleolytic attack on the DNA backbone can also remove the protein adduct from the DSB but genetic information is lost from the ends. Accurate repair of this break would therefore need HR to copy the missing information from the sister chromatid, while NHEJ would result in error-prone repair.

In summary, based on these and our previously published data, we suggest that TDP2 defines a novel error-free NHEJ sub-pathway that converts TOP2-linked 5′-termini into ligatable DNA termini. We suggest that this may be particularly important during G1 and in post-mitotic cells, which lack HR-mediated repair, and thus in which it may be the only mechanism for error-free DSB repair of TOP2-induced DSBs (Figure 8).

### TDP2 and cancer therapy

The results presented here can have important implications in the treatment of cancer. Given the widespread use of TOP2 poisons in cancer therapy, and the observed hypersensitivity to TOP2 poisons of cells lacking TDP2, our findings suggest that TDP2 could affect the response of tumour cells to chemotherapy. In this context, TDP2 expression is reportedly elevated in the majority of non-small cell lung cancer cells [43], and mutant-p53-dependent over-expression of TDP2 has been implicated in cellular resistance to etoposide in lung cancer cells [44]. TDP2 might therefore be a valid target for overcoming tumour resistance to TOP2 poisons and/or a useful predictive biomarker for clinical response to these agents.

In addition, our toxicity assays in mice and the increased genome instability in cells and in mouse bone marrow correlate well with known side effects of treatment with TOP2 poisons during cancer therapy. This raises the possibility that heterogeneity in expression levels or activity of TDP2 could be an important etiological factor both in the toxicity that accompanies chemotherapy involving TOP2 poisons [45] and on the incidence of treatment-related hematological malignancy, typically acute leukemia occurring in a relatively high proportion of patients [4], [7], [8]. Like other acute leukemias, therapy-related malignancies are linked to specific translocations that result in the expression of fusion proteins and contribute in some way to disease development. Intriguingly, in some cases, these translocations map to regions of preferential TOP2 cleavage, supporting a model in which the translocations arise via erroneous repair of TOP2-induced DSBs. These translocations are also surprisingly similar to those found in infant leukemia [46], suggesting that erroneous repair of TOP2-induced DSBs may also be a source of primary malignancy. Consistent with this idea, TOP2-induced DSBs are implicated in translocations commonly associated with prostate cancer [47]. In the light of our findings, it is tempting to speculate that TDP2 activity reduces the likelihood of oncogenic translocations, by ensuring rapid and accurate repair of TOP2-induced DSBs. It is possible, however, that TDP2 might occasionally promote a translocation, by liberating a DSB that engages in erroneous DNA ligation, as might be the case in some extremely conservative rearrangements that have been reported [12], [13].

### Conclusions

We have shown that TDP2 protects mouse cells from the cytotoxic and clastogenic effects of TOP2 poisons, most likely by functioning in error-free pathway for NHEJ. These results have important implications in the treatment of cancer. For example, development of small molecule inhibitors for TDP2 may provide a way of sensitizing particular types of tumor to chemotherapy, though precaution is necessary to consider the possible consequences of TDP2 inhibition on normal cells and on the generation of secondary malignancies.

## Materials and Methods

### Ethics statement

All animal procedures were performed in accordance with European Union legislation and with the approval of the Ethical Committee for Animal Experimentation of the University of Leuven and the University of Seville, respectively.

### Cells and cell culture

Chicken DT40 B lymphoma cells were cultured at 39°C, 5% CO_2_ in RPMI 1640 medium supplemented with 10^−5^ M β-mercaptoethanol, penicillin, streptomycin, 10% fetal calf serum (FCS), and 1% chicken serum (Sigma). *TDP2^−/−/−^* cell line was previously described [25]. To generate *KU70* deletion constructs, Hygromycin (Hygro^R^) or Neomycin (Neo^R^) resistance cassettes were inserted between sequences of 1.6 kb and 3.3 kb in length from the *KU70* locus [48]. KU70-Hygro^R^ and KU70-Neo^R^ deletion constructs were sequentially transfected into wild-type and *TDP2^−/−/−^* cells. The gene targeting events were confirmed by Southern blot analysis of *EcoRI* -digested genomic DNA hybridized to an external probe (Figure S1).

Transformed human fibroblast lines 1BR (wild-type) and HSC62 (BRCA2-mutant) were described previously [49]. Cells were cultured in DMEM supplemented with penicillin, streptomycin and 15% FCS.

Primary MEFs were isolated from littermate embryos at day 13 p.c. and cultured at 37°C, 5% CO_2_, 3% O_2_ in Dubelcco's Modified Eagle's Medium (DMEM) supplemented with penicillin, streptomycin, 10% FCS and non-essential aminoacids. All experiments were carried out between P2 and P4. MEFs were transformed by retroviral delivery of T121, a fragment of the SV40 large T antigen that antagonizes the three Rb family members but not p53 [50]. Transformed MEFs were maintained at 37°C, 5% CO_2_ in DMEM supplemented with penicillin, streptomycin and 10% FCS.

### Generation of Tdp2 conditional and knockout mice

A targeting construct was generated for *Tdp2* in which the first three exons were flanked by *lox*P sites, followed by an FRT- and *lox*P-flanked neomycin-resistance (neo) cassette. These three exons encode for the N-terminal half of TDP2 and contain mapped interaction domains for e.g. TDP2 itself, CD40 and TRAF6 [23]. The *Tdp2^flEx1–3,neo^* targeting construct was electroporated in E14 (129Ola) ES cells and correctly recombined ES cell clones were confirmed by Southern blot analysis. The functionality of the *lox*P sites was shown *in vitro* by electroporation of a correctly targeted ES cell clone with a Cre-expressing vector. Several correctly targeted ES cell clones were used for aggregation with CD1 morulae and transferred into pseudo-pregnant recipient females to obtain chimaeric mice. Three chimaeric males produced heterozygous offspring after breeding with CD1 wild-type females. The obtained offspring was genotyped with both a *lox*P-specific and a neo-specific PCR. Intercrosses between *Tdp2^flEx1–3,neo/+^* mice led to the generation of homozygous floxed *Tdp2* mice which were viable and fertile. To delete the critical exons we crossed the heterozygous *Tdp2* mice with an EIIa-Cre mouse (Adenovirus EIIa-promoter driven Cre) and obtained *Tdp2^flΔ/+^* mice. Intercrosses of the latter mice resulted in viable homozygous knockout mice (from now on denoted *Tdp2^Δ1–3^*) at the normal 25% Mendelian distribution. Southern blot analysis confirmed the complete recombination of the *lox*P-flanked sequences in the homozygous mice and hence the generation of *Tdp2* knockout mice.

### 5′-TDP activity *in vitro*

Labelled double-stranded 5′-phosphotyrosyl substrates were generated essentially as previously described [22], [22,25]. For 5′ overhang substrates 5′-Y-P-AATTCTTCTCTTTCCAGGGCTATGT-3′ (Midland Certified Reagents) and 5′-AGACATAGCCCTGGAAAGAGAAG-3′ (Sigma) oligonucleotides were annealed. Cell and tissue extracts were prepared by mild sonication in Lysis Buffer (40 mM Tris–HCl, pH 7.5, 100 mM NaCl, 0.1% Tween-20, 1 mM DTT) supplemented with 1 mM PMSF and protease inhibitor cocktail (Sigma) and clarification by centrifugation 10 min 16500 g 4°C. Protein concentration was measured with Bradford reagent (Sigma). 5′-TDP reactions contained 50 nM substrate, 80 µM competitor single-stranded oligonucleotide and the indicated amount of protein extract in a total volume of 6 µl Reaction Buffer (50 mM Tris-Cl, pH 7.5, 50 mM KCl, 1 mM MgCl_2_, 1 mM DTT, 100 µg/ml BSA). Ligation reactions with oligos contained in addition 5 units of T4 DNA ligase (Fermentas) and 1 mM ATP (Sigma) Reactions were stopped by the addition of 3 µl 3× Formamide Loading Buffer and 5 min 95°C incubation. Samples were resolved by denaturing polyacrylamide gel electrophoresis and analysed by phosphorimaging in a Fujifilm FLA5100 device (GE Healthcare).

### Plasmid circularization assays

Substrates were generated by PCR-mediated amplification of plasmid pEGFP-Pem1-Ad2 [51] with primers 5′-AATTCTTCTCTTTCCAGGGCTATGT-3′ and 5′- AATTCATCCCCAGAAATGTAACTTG-3′ harbouring phosphate (Sigma) or phosphotyrosine (Midland Certified Reagents) moieties at 5′ ends. NHEJ-competent nuclear extracts were prepared as previously described [28]. Reactions were performed by incubating (6 h at 16°C) 100 ng of each substrate with 7 µg of nuclear extracts in NHEJ Buffer (50 mM Tris-HCl pH 7.5, 50 mM KCl, 1 mM DTT, 2 mM MgCl2, 1 mM ATP, 100 µg/ml BSA) in the presence or absence of 50 nM hTDP2 recombinant protein (purified as previously described [22]). Reactions were stopped by addition of EDTA (to a final concentration of 100 mM) and treated 30′ with Proteinase K (0.2 mg/ml). DNA was purified using FavorPrep GEL/PCR Purification Mini Kit (Favorgen) and transformed into MegaX DH10B T1 Electrocompetent Cells (Invitrogen). Positive transformants were selected by plating on LB agar plates containing kanamycin (25 µg/ml).

### Clonogenic survival assays

To determine sensitivity in DT40, cells were plated in 5 ml of medium containing 1.5% (by weight) methylcellulose (Sigma) in 6-well plates at 50, 500, and 5000 cells/well per treatment condition. Media also contained the indicated concentration of doxorubicin (Sigma), mAMSA (Sigma) or etoposide (Sigma). In all experiments, cells were incubated for 7–11 days and visible colonies were counted.

Survival assays in MEFs were carried out seeding 2000 cells in 100 mm dishes, in duplicate for each experimental condition. After 6 hours, cells were irradiated or treated with the indicated concentrations of etoposide for 3 hours, washed with PBS and fresh medium was added. Cells were incubated for 10–14 days and fixed and stained for colony scoring in Crystal violet solution (0.5% Crystal violet in 20% ethanol). The surviving fraction at each dose was calculated by dividing the average number of visible colonies in treated versus untreated dishes.

Human fibroblasts were transfected with non-targeting Negative Control and *TDP2* smartpool siRNAs (Thermo Scientific) using HyperFect transfection reagent (Invitrogen). Cells were transfected twice in two consecutive days and used for survival 48 hours after second transfection. Other details as described above.

### Immunofluorescence and antibodies

MEFs grown on coverslips for the required time, 7 days for confluency-arrested cells and 2 days for cycling cells, were treated as indicated and fixed (10 min in PBS-4% paraformaldehyde), permeabilized (2 min in PBS-0.2% Triton X-100), blocked (30 min in PBS-5% BSA) and incubated with the required primary antibodies (1–3 h in PBS-1% BSA). Cells were then washed (3×5 min in PBS-0.1% Tween 20), incubated for 30 min with the corresponding AlexaFluor-conjugated secondary antibodies (1/1000 dilution in 1% BSA-PBS) and washed again as described above. Finally, they were counterstained with DAPI (Sigma) and mounted in Vectashield (Vector Labs). Rad51 foci scoring requires 30 sec. pre-extraction in PBS-0.1% Triton X-100 prior to fixation. γH2AX and Rad51 foci were manually counted (double-blind) in 40 cells from each experimental condition. When necessary to identify replicating cells, 5-ethynyl-2′-deoxyuridine (EdU, Invitrogen) was added throughout treatment and repair at a final concentration of 10 µM. Click chemistry reaction was performed before DAPI staining by incubating (30 min r.t.) with 1 µM AlexaFluor-conjugated azide (Invitrogen) in reaction cocktail (100 mM TrisHCl pH 8.5, 1 mM CuSO_4_, 100 mM ascorbic acid). For the analysis of G0/G1 confluency-arrested cells only Cyclin A negative cells were scored. For G2, as EdU was present (10 µM) during and after treatment, only Cyclin A positive cells without EdU incorporation were scored (see Figure S3). Primary antibodies were used at the indicated dilution: γH2AX (Millipore, 05-636) 1/1000, Cyclin A (Santa Cruz, sc-751) 1/500, Rad51 (Abcam, ab213) 1/200 and Tubulin (Santa Cruz, sc-5286) 1/2500.

### Cytogenetic analysis

Micronuclei and nucleoplasmic bridges were analysed in transformed and low passage primary MEFs previously seeded onto coverslips. Following treatment, cytochalasin B (Sigma) was added at 4 µg/ml to transformed but not to primary MEFs. 22 h (transformed) or 30 h (primary) post-treatment, cells were fixed and subject to DAPI staining as described above. In transformed cells only binucleated cells were scored, which was confirmed by visualization of the cytoplasm with anti Tubulin immunofluorescence (performed as described above).

Chromosomal aberrations were scored in Giemsa stained metaphase spreads. Following treatment, recovery in fresh medium was allowed for 2 h (transformed MEFs) or 4 h (primary MEFs) and demecolcine (Sigma) was added at a final concentration of 0.2 µg/ml. Caffeine (Sigma) was also added at a final concentration of 0.1 µg/ml but only to primary cells. 4 h later cells were collected by trypsinisation, subject to hypotonic shock for 1 hour at 37°C in 0.3 M sodium citrate and fixed in 3∶1 methanol∶acetic acid solution. Cells were dropped onto acetic acid humidified slides and stained 20 minutes in Giemsa-modified (Sigma) solution (5% v/v in H_2_O).

For SCEs 10 µM BrdU (Sigma) was added to the medium for two complete cycles (approximately 48 hours) before collection. Drug treatment was applied for 30 minutes 6–8 hours before cell collection. Metaphase spreads were obtained as described above. Before Giemsa staining, slides were incubated in Hoescht 33258 solution (10 µg/ml) for 20 minutes, exposed to UV light (355 nm) for 1 hour and washed for 1 hour at 60°C in 20× SCC.

### Animal maintenance

The mouse colony was maintained in an outbred 129Ola, CD1 and C57BL/6 background under standard housing conditions, at 21±1°C with a photoperiod of 12∶12 h (lights on at 8:00). They were housed in isolated cages with controlled ventilation trough HEPA-filters and were in flow cabins. Sterile food pellets and water were available *ad libitum*. Breeding pairs between heterozygotes (*Tdp2^+/flΔ^*×*Tdp2^+/flΔ^*) were set to obtain wild-type (*Tdp2^+/+^*) and knock-out (*Tdp2^Δ1–3^*) littermates for analysis. Mice were genotyped with Phire Animal Tissue Direct PCR Kit (Thermo) following manufacturer instructions and using primers 5′-CCTTCATTACTTCTCGTAGGTTCTGGGTC-3′, 5′-ACCCGCTCTTCACGCTGCTTCC-3′ and 5′-TACACCGTGCCATAATGACCAAC-3′. This results in amplification of a 429 bp fragment from the wild-type allele or 561 bp fragment from the mutant allele.

### *In vivo* etoposide sensitivity

At 8 weeks of age, *Tdp2^+/+^* and *Tdp2^Δ1–3^* mice underwent intraperitoneal injection with 3 µl/g of body weight of either DMSO (vehicle control) or etoposide at 25 mg/ml in DMSO for a final dose of 75 mg/kg. Weight and general health status was monitored daily from the day of injection (inclusive). 6 days post-treatment mice were sacrificed by cervical dislocation and dissected for histopathological analysis. Weight of spleen and thymus was recorded prior to their histological or cell content analysis. Bone marrow (BM) from femurs and tibias of each mouse was also obtained.

For histological analysis organs were fixed in 4% paraformaldehyde for 2 days, embedded in paraffin, cut in 6 µm slices by microtome, stained with Hematoxylin-Eosin and visualized under the microscope. For cell content analysis by FACS, BM and thymus were homogenized in EDTA Buffer (140 mM NaCL, 1.5 mM KH_2_PO_4_, 2.7 mM KCl, 8.1 mM Na_2_HPO_4_, 0.6 mM EDTA). Cells from both tissues were immunolabelled with the appropriate fluorescently-labelled antibodies according to manufacture's recommendations and analyzed using a FACScalibur flow cytometer (Becton Dickinson): B220-APC (17-0452), CD43 FITC (11-0431), CD8 APC (17-0081) and CD4 FITC (11-0043) (eBiosciences); CD11b/Mac-1 PE (550019) (Becton Dickinson). Data was compiled and analysed using CellQuest software (Becton Dickinson).

### Micronuclei analysis *in vivo*

At 8 weeks of age, *Tdp2^+/+^* and *Tdp2^Δ1–3^* mice underwent intraperitoneal injection with 2.5 µl/g of body weight of either 10% DMSO (vehicle control) or etoposide at 400 µg/ml in 10% DMSO for a final dose of 1 mg/kg. Mice were sacrificed by cervical dislocation 24 h after injection and BM from one femur and tibia was extracted and homogenized in 3 ml FBS. Cellular content was concentrated in 150 µl FBS by centrifugation and smears were prepared on glass slides. Following 5 min fixation in methanol, slides were stained 30 min in Giemsa-modified (Sigma) solution (5% v/v in 100 mM Tris-HCl pH 6.8) and visualized under the microscope. 2000 polychromatic erythrocytes (PCE) were scored for the presence of micronuclei (MN-PCE) in each slide.

## Supporting Information

Figure S1 TDP2 promotes survival following TOP2-induced DSBs in mammalian cells. Clonogenic survival of wild-type and Tdp2Δ1–3 transformed MEFs after continuous exposure to the indicated concentrations of etoposide. Average ± s.e.m. of three independent experiments and statistical significance by Two-way ANOVA test with Bonferroni post-test is shown.
(TIF)

Figure S2 Targeted deletion of *KU70* in DT40 cells. Southern-blot analysis of *EcoRI*-digested DNA from wild-type (*+/+*), heterozygous (*+/−*) and knock-out (*−/−*) DT40 cells in *TDP2^+/+/+^* and *TDP2^−/−/−^* background. A probe hybridizing to a region of the *KU70* locus not contained in the deletion construct was used. The 5.5-kb (wild-type) and 2.8-kb (deleted) expected bands are indicated. Note that two clones were selected for further analysis.
(TIF)

Figure S3 Cell cycle dependent induction of DSBs following etoposide treatment in primary MEFs. DSBs detected as γH2AX foci (bottom left), Cyclin A content (Cyc A, top right), 5-ethynyl-2′-deoxyuridine incorporation (EdU, bottom right) and DAPI counterstain (top left) are shown. G1 (Cyc A negative, EdU negative), S-phase (Cyc A positive, EdU positive) and G2 (Cyc A positive, EdU positive) nuclei are indicated (arrows).
(TIF)

Figure S4 TDP2 depletion impairs repair of TOP2-induced DSBs in human A549 cells. A. γH2AX foci induction after 30 min 50 µM etoposide treatment and repair at different times following drug removal in G1 TDP2-depleted (pS-TDP2) and control non-depleted (pS) cells. B. G2 cells with 10 µM etoposide treatment. Other details as in “A”. Average ± s.e.m. of at least three independent experiments is shown. Statistical significance by Two-way ANOVA test with Bonferroni post-test is indicated. TDP2 depletion was performed as previously described [22].
(TIF)

Figure S5 The absence of TDP2 sensitizes lymphocyte precursors to etoposide treatment *in vivo*. FACS analysis of B-cells in bone marrow (A) and T-cells in thymus (B) in wild-type and *Tdp2^Δ1–3^* animals 6 days after treatment with 75 mg/kg etoposide or vehicle control (DMSO). Pro-B cell (CD43^+^ B220^+^), Pre-B cell (CD43^−^ B220^low^) immature B cell (CD43^−^ B220^high^) and immature T cell (CD4^+^ CD8^+^) populations are indicated (rectangles).
(TIF)

Figure S6 Myeloid cells are not significantly affected by etoposide treatment. FACS analysis of Mac1^+^ myeloid cells in bone marrow in wild-type and *Tdp2^Δ1–3^* animals 6 days after treatment with 75 mg/kg etoposide or vehicle control (DMSO). Scatter plot (A) and quantification (B) are shown. The mild increase correlates with the observed decrease in lymphocite precursors.
(TIF)

## References

[1] Champoux JJ (2001) DNA topoisomerases: structure, function, and mechanism. Annu Rev Biochem 70: 369–413 doi:10.1146/annurev.biochem.70.1.369. 11395412 10.1146/annurev.biochem.70.1.369

[2] Wang JC (2002) Cellular roles of DNA topoisomerases: a molecular perspective. Nat Rev Mol Cell Biol 3: 430–440 doi:10.1038/nrm831. 12042765 10.1038/nrm831

[3] Nitiss JL (2009) DNA topoisomerase II and its growing repertoire of biological functions. Nat Rev Cancer 9: 327–337 doi:10.1038/nrc2608. 19377505 10.1038/nrc2608 PMC2730144

[4] Deweese JE, Osheroff N (2009) The DNA cleavage reaction of topoisomerase II: wolf in sheep's clothing. Nucleic Acids Res 37: 738–748 doi:10.1093/nar/gkn937. 19042970 10.1093/nar/gkn937 PMC2647315

[5] Nitiss JL (2009) Targeting DNA topoisomerase II in cancer chemotherapy. Nat Rev Cancer 9: 338–350 doi:10.1038/nrc2607. 19377506 10.1038/nrc2607 PMC2748742

[6] Anderson RD, Berger NA (1994) International Commission for Protection Against Environmental Mutagens and Carcinogens. Mutagenicity and carcinogenicity of topoisomerase-interactive agents. Mutat Res 309: 109–142. 7519727 10.1016/0027-5107(94)90048-5

[7] Felix CA, Kolaris CP, Osheroff N (2006) Topoisomerase II and the etiology of chromosomal translocations. DNA Repair (Amst) 5: 1093–1108 doi:10.1016/j.dnarep.2006.05.031. 16857431 10.1016/j.dnarep.2006.05.031

[8] Povirk LF (2006) Biochemical mechanisms of chromosomal translocations resulting from DNA double-strand breaks. DNA Repair (Amst) 5: 1199–1212 doi:10.1016/j.dnarep.2006.05.016. 16822725 10.1016/j.dnarep.2006.05.016

[9] Albain KS, Le Beau MM, Ullirsch R, Schumacher H (1990) Implication of prior treatment with drug combinations including inhibitors of topoisomerase II in therapy-related monocytic leukemia with a 9;11 translocation. Gene Chromosome Canc 2: 53–58. 10.1002/gcc.2870020110 2177642

[10] Ahuja HG, Felix CA, Aplan PD (2000) Potential role for DNA topoisomerase II poisons in the generation of t(11;20)(p15;q11) translocations. Gene Chromosome Canc 29: 96–105. 10.1002/1098-2264(2000)9999:9999<::aid-gcc1013>3.0.co;2-t 10959088

[11] Andersen MK, Christiansen DH, Jensen BA, Ernst P, Hauge G, et al (2001) Therapy-related acute lymphoblastic leukaemia with MLL rearrangements following DNA topoisomerase II inhibitors, an increasing problem: report on two new cases and review of the literature since 1992. Br J Haematol 114: 539–543. 11552977 10.1046/j.1365-2141.2001.03000.x

[12] Lovett BD, Lo Nigro L, Rappaport EF, Blair IA, Osheroff N, et al (2001) Near-precise interchromosomal recombination and functional DNA topoisomerase II cleavage sites at MLL and AF-4 genomic breakpoints in treatment-related acute lymphoblastic leukemia with t(4;11) translocation. Proc Natl Acad Sci USA 98: 9802–9807 doi:10.1073/pnas.171309898. 11493704 10.1073/pnas.171309898 PMC55533

[13] Whitmarsh RJ, Saginario C, Zhuo Y, Hilgenfeld E, Rappaport EF, et al (2003) Reciprocal DNA topoisomerase II cleavage events at 5“-TATTA-3” sequences in MLL and AF-9 create homologous single-stranded overhangs that anneal to form der(11) and der(9) genomic breakpoint junctions in treatment-related AML without further processing. Oncogene 22: 8448–8459 doi:10.1038/sj.onc.1207052. 14627986 10.1038/sj.onc.1207052

[14] Mistry AR, Felix CA, Whitmarsh RJ, Mason A, Reiter A, et al (2005) DNA topoisomerase II in therapy-related acute promyelocytic leukemia. N Engl J Med 352: 1529–1538 doi:10.1056/NEJMoa042715. 15829534 10.1056/NEJMoa042715

[15] Giguère A, Hébert J (2011) Microhomologies and topoisomerase II consensus sequences identified near the breakpoint junctions of the recurrent t(7;21)(p22;q22) translocation in acute myeloid leukemia. Gene Chromosome Canc 50: 228–238 doi:10.1002/gcc.20848. 10.1002/gcc.20848 21319259

[16] Le H, Singh S, Shih S-J, Du N, Schnyder S, et al (2009) Rearrangements of the MLL gene are influenced by DNA secondary structure, potentially mediated by topoisomerase II binding. Gene Chromosome Canc 48: 806–815 doi:10.1002/gcc.20685. 10.1002/gcc.20685 PMC2764312 19530238

[17] Mirault M-E, Boucher P, Tremblay A (2006) Nucleotide-resolution mapping of topoisomerase-mediated and apoptotic DNA strand scissions at or near an MLL translocation hotspot. Am J Hum Genet 79: 779–791 doi:10.1086/507791. 17033956 10.1086/507791 PMC1698565

[18] Connelly JC, Leach DRF (2004) Repair of DNA covalently linked to protein. Mol Cell 13: 307–316. 14967139 10.1016/s1097-2765(04)00056-5

[19] Kurosawa A, Koyama H, Takayama S, Miki K, Ayusawa D, et al (2008) The requirement of Artemis in double-strand break repair depends on the type of DNA damage. DNA Cell Biol 27: 55–61 doi:10.1089/dna.2007.0649. 17941805 10.1089/dna.2007.0649

[20] Sartori AA, Lukas C, Coates J, Mistrik M, Fu S, et al (2007) Human CtIP promotes DNA end resection. Nature 450: 509–514 doi:10.1038/nature06337. 17965729 10.1038/nature06337 PMC2409435

[21] Quennet V, Beucher A, Barton O, Takeda S, Löbrich M (2011) CtIP and MRN promote non-homologous end-joining of etoposide-induced DNA double-strand breaks in G1. Nucleic Acids Res 39: 2144–2152 doi:10.1093/nar/gkq1175. 21087997 10.1093/nar/gkq1175 PMC3064790

[22] Cortes Ledesma F, Khamisy El SF, Zuma MC, Osborn K, Caldecott KW (2009) A human 5′-tyrosyl DNA phosphodiesterase that repairs topoisomerase-mediated DNA damage. Nature 461: 674–678 doi:10.1038/nature08444. 19794497 10.1038/nature08444

[23] Pype S, Declercq W, Ibrahimi A, Michiels C, Van Rietschoten JG, et al (2000) TTRAP, a novel protein that associates with CD40, tumor necrosis factor (TNF) receptor-75 and TNF receptor-associated factors (TRAFs), and that inhibits nuclear factor-kappa B activation. J Biol Chem 275: 18586–18593 doi:10.1074/jbc.M000531200. 10764746 10.1074/jbc.M000531200

[24] Pei H, Yordy JS, Leng Q, Zhao Q, Watson DK, et al (2003) EAPII interacts with ETS1 and modulates its transcriptional function. Oncogene 22: 2699–2709 doi:10.1038/sj.onc.1206374. 12743594 10.1038/sj.onc.1206374

[25] Zeng Z, Cortes Ledesma F, Khamisy El SF, Caldecott KW (2011) TDP2/TTRAP is the major 5′-tyrosyl DNA phosphodiesterase activity in vertebrate cells and is critical for cellular resistance to topoisomerase II-induced DNA damage. J Biol Chem 286: 403–409 doi:10.1074/jbc.M110.181016. 21030584 10.1074/jbc.M110.181016 PMC3012998

[26] Shrivastav M, De Haro LP, Nickoloff JA (2008) Regulation of DNA double-strand break repair pathway choice. Cell Res 18: 134–147 doi:10.1038/cr.2007.111. 18157161 10.1038/cr.2007.111

[27] Murai J, Huang S-YN, Das BB, Dexheimer TS, Takeda S, et al (2012) Tyrosyl-DNA phosphodiesterase 1 (TDP1) repairs DNA damage induced by topoisomerases I and II and base alkylation in vertebrate cells. J Biol Chem 287: 12848–12857 doi:10.1074/jbc.M111.333963. 22375014 10.1074/jbc.M111.333963 PMC3339927

[28] Baumann P, West SC (1998) DNA end-joining catalyzed by human cell-free extracts. Proc Natl Acad Sci USA 95: 14066–14070. 9826654 10.1073/pnas.95.24.14066 PMC24327

[29] Löbrich M, Shibata A, Beucher A, Fisher A, Ensminger M, et al (2010) gammaH2AX foci analysis for monitoring DNA double-strand break repair: strengths, limitations and optimization. Cell Cycle 9: 662–669. 20139725 10.4161/cc.9.4.10764

[30] Huertas P (2010) DNA resection in eukaryotes: deciding how to fix the break. Nat Struct Mol Biol 17: 11–16 doi:10.1038/nsmb.1710. 20051983 10.1038/nsmb.1710 PMC2850169

[31] Sonoda E, Sasaki MS, Morrison C, Yamaguchi-Iwai Y, Takata M, et al (1999) Sister chromatid exchanges are mediated by homologous recombination in vertebrate cells. Mol Cell Biol 19: 5166–5169. 10373565 10.1128/mcb.19.7.5166 PMC84359

[32] Takahashi N, Kadota T, Kawano S, Ishikawa K, Kuroyanagi K, et al (1986) [Toxicity studies of VP 16-213 (I)–Acute toxicity in mice, rats and rabbits]. J Toxicol Sci 11 Suppl 1: 1–16. 10.2131/jts.11.supplementi_1 3761389

[33] Li C, Sun S-Y, Khuri FR, Li R (2011) Pleiotropic functions of EAPII/TTRAP/TDP2: cancer development, chemoresistance and beyond. Cell Cycle 10: 3274–3283 doi:10.4161/cc.10.19.17763. 21926483 10.4161/cc.10.19.17763 PMC3233625

[34] Barthelmes HU, Habermeyer M, Christensen MO, Mielke C, Interthal H, et al (2004) TDP1 overexpression in human cells counteracts DNA damage mediated by topoisomerases I and II. J Biol Chem 279: 55618–55625 doi:10.1074/jbc.M405042200. 15494395 10.1074/jbc.M405042200

[35] Nitiss KC, Malik M, He X, White SW, Nitiss JL (2006) Tyrosyl-DNA phosphodiesterase (Tdp1) participates in the repair of TOP2–mediated DNA damage. Proc Natl Acad Sci USA 103: 8953–8958 doi:10.1073/pnas.0603455103. 16751265 10.1073/pnas.0603455103 PMC1482547

[36] Zeng Z, Sharma A, Ju L, Murai J, Umans L, et al (2012) TDP2 promotes repair of topoisomerase I-mediated DNA damage in the absence of TDP1. Nucleic Acids Res doi:10.1093/nar/gks622. 10.1093/nar/gks622 PMC3458563 22740648

[37] Symington LS, Gautier J (2011) Double-strand break end resection and repair pathway choice. Annu Rev Genet 45: 247–271 doi:10.1146/annurev-genet-110410-132435. 21910633 10.1146/annurev-genet-110410-132435

[38] de Campos-Nebel M, Larripa I, González-Cid M (2010) Topoisomerase II-mediated DNA damage is differently repaired during the cell cycle by non-homologous end joining and homologous recombination. PLoS ONE 5: e12541 doi:10.1371/journal.pone.0012541. 20824055 10.1371/journal.pone.0012541 PMC2932731

[39] Li GC, Ouyang H, Li X, Nagasawa H, Little JB, et al (1998) Ku70: a candidate tumor suppressor gene for murine T cell lymphoma. Mol Cell 2: 1–8. 9702186 10.1016/s1097-2765(00)80108-2

[40] Pierce AJ, Hu P, Han M, Ellis N, Jasin M (2001) Ku DNA end-binding protein modulates homologous repair of double-strand breaks in mammalian cells. Genes Dev 15: 3237–3242 doi:10.1101/gad.946401. 11751629 10.1101/gad.946401 PMC312854

[41] Saintigny Y, Delacôte F, Boucher D, Averbeck D, Lopez BS (2007) XRCC4 in G1 suppresses homologous recombination in S/G2, in G1 checkpoint-defective cells. Oncogene 26: 2769–2780 doi:10.1038/sj.onc.1210075. 17057732 10.1038/sj.onc.1210075

[42] Cortes Ledesma F, Prado F, Aguilera A (2007) Sister chromatid recombination. In: Aguilera A, Rothstein R, editors. Top Curr Genet 17 (Molecular Genetics of Recombination). Berlin Heidelberg: Springer-Verlag. pp. 363–380.

[43] Li C, Fan S, Owonikoko TK, Khuri FR, Sun S-Y, et al (2011) Oncogenic role of EAPII in lung cancer development and its activation of the MAPK-ERK pathway. Oncogene 30: 3802–3812 doi:10.1038/onc.2011.94. 21478903 10.1038/onc.2011.94 PMC3220271

[44] Do PM, Varanasi L, Fan S, Li C, Kubacka I, et al (2012) Mutant p53 cooperates with ETS2 to promote etoposide resistance. Genes Dev 26: 830–845 doi:10.1101/gad.181685.111. 22508727 10.1101/gad.181685.111 PMC3337457

[45] Wolff SN, Hainsworth JD, Greco FA (2008) High-dose etoposide: from phase I to a component of curative therapy. J Clin Oncol 26: 5310–5312 doi:10.1200/JCO.2008.19.0892. 18838698 10.1200/JCO.2008.19.0892 PMC2661465

[46] Langer T, Metzler M, Reinhardt D, Viehmann S, Borkhardt A, et al (2003) Analysis of t(9;11) chromosomal breakpoint sequences in childhood acute leukemia: almost identical MLL breakpoints in therapy-related AML after treatment without etoposides. Gene Chromosome Canc 36: 393–401 doi:10.1002/gcc.10167. 10.1002/gcc.10167 12619163

[47] Haffner MC, Aryee MJ, Toubaji A, Esopi DM, Albadine R, et al (2010) Androgen-induced TOP2B-mediated double-strand breaks and prostate cancer gene rearrangements. Nat Genet 42: 668–675 doi:10.1038/ng.613. 20601956 10.1038/ng.613 PMC3157086

[48] Takata M, Sasaki MS, Sonoda E, Morrison C, Hashimoto M, et al (1998) Homologous recombination and non-homologous end-joining pathways of DNA double-strand break repair have overlapping roles in the maintenance of chromosomal integrity in vertebrate cells. EMBO J 17: 5497–5508 doi:10.1093/emboj/17.18.5497. 9736627 10.1093/emboj/17.18.5497 PMC1170875

[49] Beucher A, Birraux J, Tchouandong L, Barton O, Shibata A, et al (2009) ATM and Artemis promote homologous recombination of radiation-induced DNA double-strand breaks in G2. EMBO J 28: 3413–3427 doi:10.1038/emboj.2009.276. 19779458 10.1038/emboj.2009.276 PMC2752027

[50] Sáenz Robles MT, Symonds H, Chen J, Van Dyke T (1994) Induction versus progression of brain tumor development: differential functions for the pRB- and p53-targeting domains of simian virus 40 T antigen. Mol Cell Biol 14: 2686–2698. 8139568 10.1128/mcb.14.4.2686-2698.1994 PMC358635

[51] Seluanov A, Mittelman D, Pereira-Smith OM, Wilson JH, Gorbunova V (2004) DNA end joining becomes less efficient and more error-prone during cellular senescence. Proc Natl Acad Sci USA 101: 7624–7629 doi:10.1073/pnas.0400726101. 15123826 10.1073/pnas.0400726101 PMC419656

