# Supplementary figures and images for: TDP2–Dependent Non-Homologous End-Joining Protects against Topoisomerase II–Induced DNA Breaks and Genome Instability in Cells and In Vivo
Source: PLoS Genet. 2013 Mar 7;9(3):e1003226. doi: 10.1371/journal.pgen.1003226 (PMC3592926; doi:10.1371/journal.pgen.1003226)

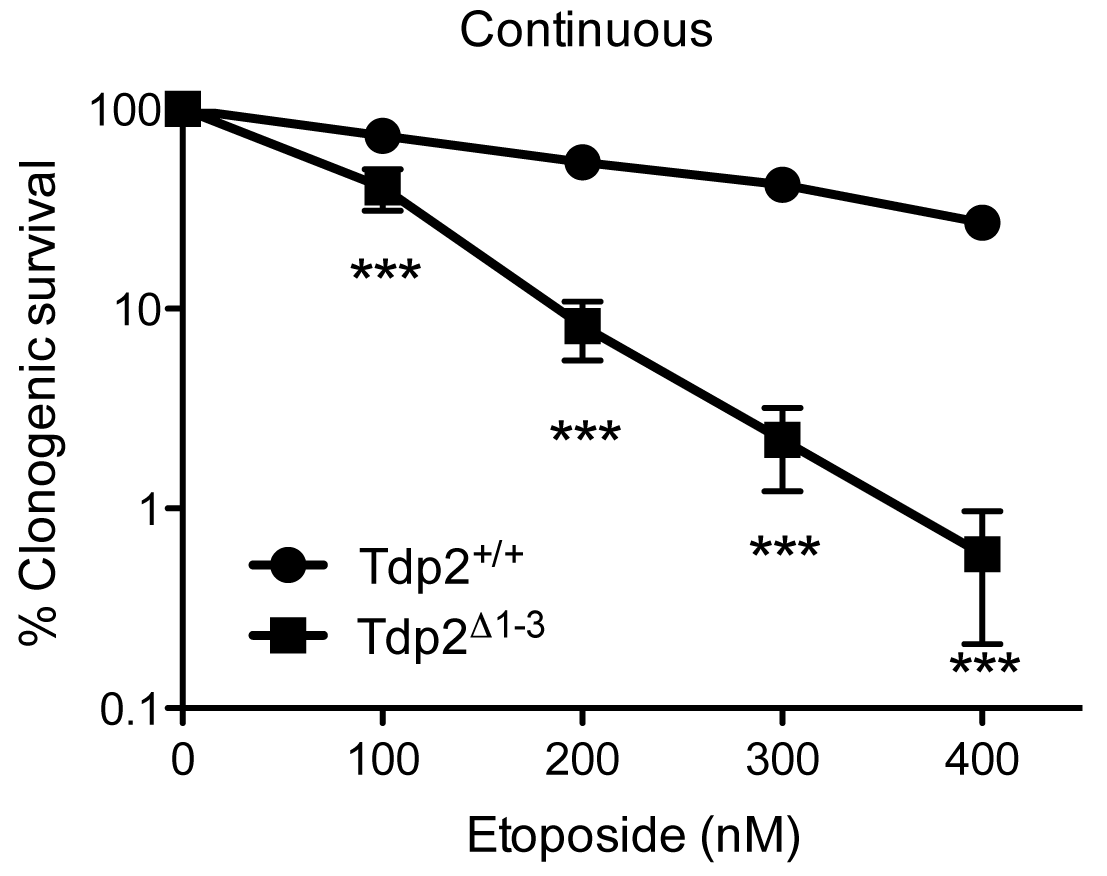

Supplement: Figure S1 — TDP2 promotes survival following TOP2-induced DSBs in mammalian cells. Clonogenic survival of wild-type and Tdp2Δ1–3 transformed MEFs after continuous exposure to the indicated concentrations of etoposide. Average ± s.e.m. of three independent experiments and statistical significance by Two-way ANOVA test with Bonferroni post-test is shown. (TIF) [file pgen.1003226.s001.tif]

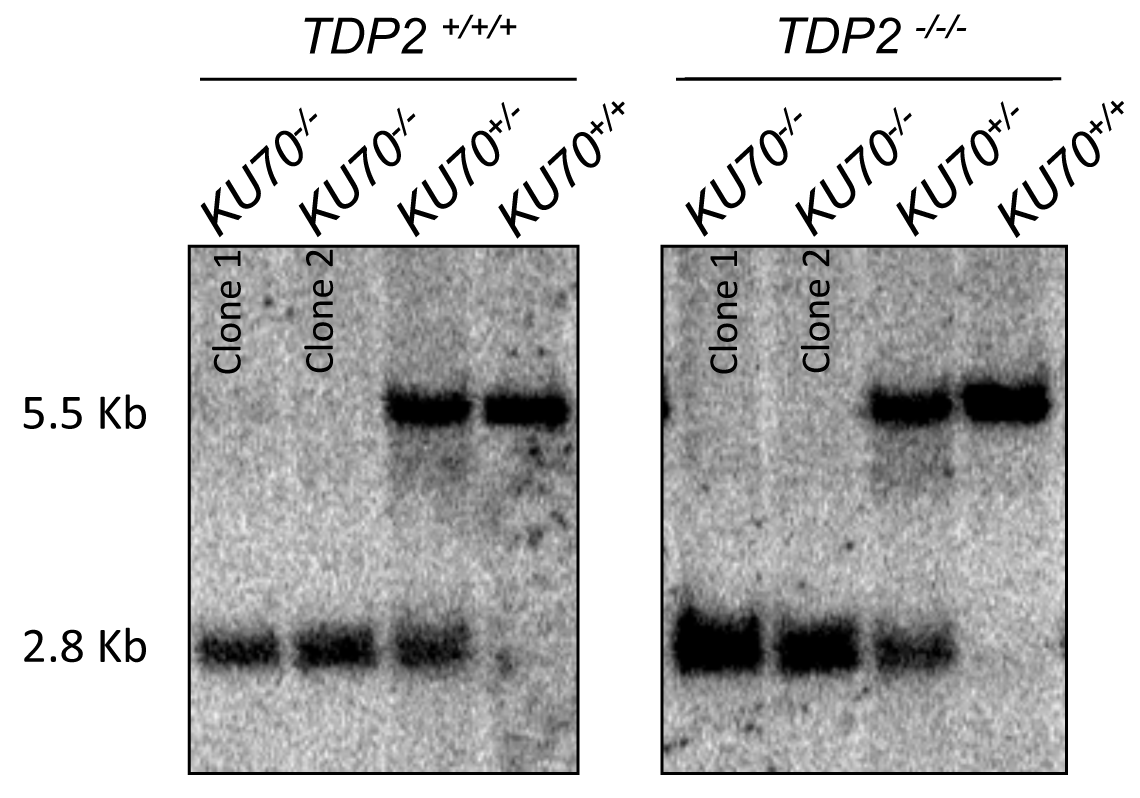

Supplement: Figure S2 — Targeted deletion of KU70 in DT40 cells. Southern-blot analysis of EcoRI-digested DNA from wild-type (+/+), heterozygous (+/−) and knock-out (−/−) DT40 cells in TDP2+/+/+ and TDP2−/−/− background. A probe hybridizing to a region of the KU70 locus not contained in the deletion construct was used. The 5.5-kb (wild-type) and 2.8-kb (deleted) expected bands are indicated. Note that two clones were selected for further analysis. (TIF) [file pgen.1003226.s002.tif]

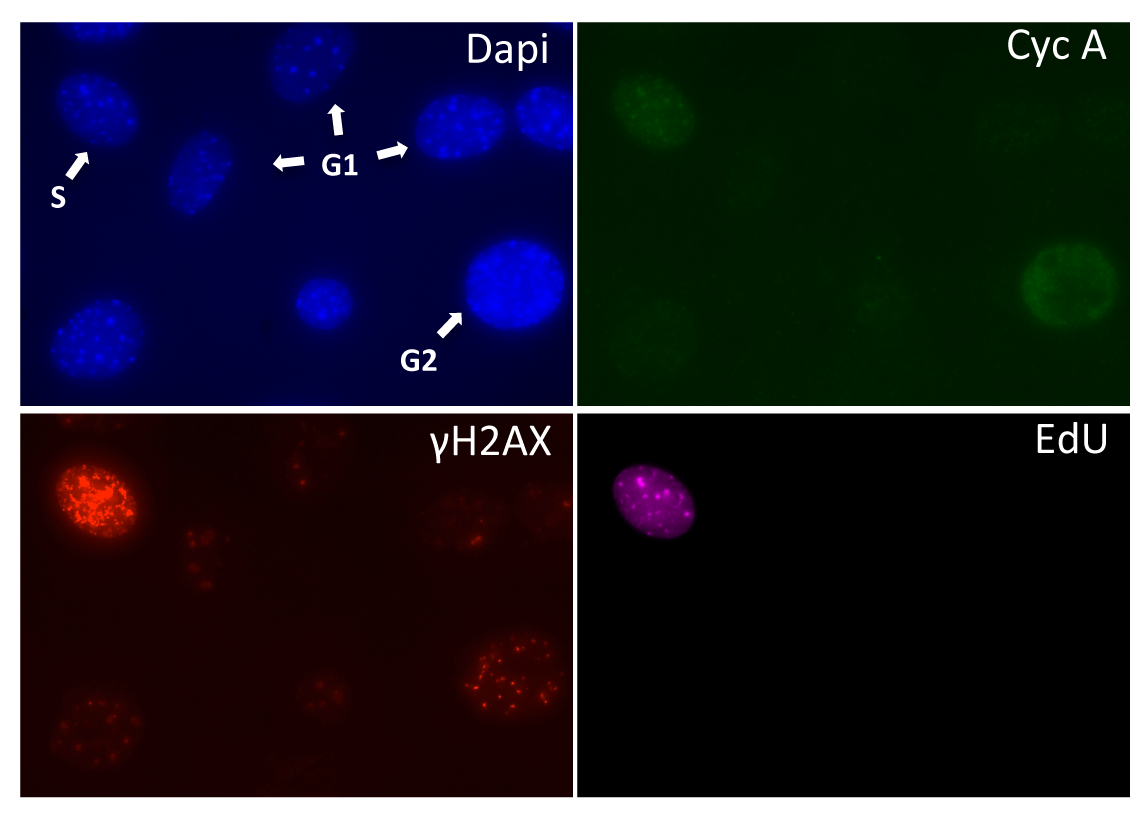

Supplement: Figure S3 — Cell cycle dependent induction of DSBs following etoposide treatment in primary MEFs. DSBs detected as γH2AX foci (bottom left), Cyclin A content (Cyc A, top right), 5-ethynyl-2′-deoxyuridine incorporation (EdU, bottom right) and DAPI counterstain (top left) are shown. G1 (Cyc A negative, EdU negative), S-phase (Cyc A positive, EdU positive) and G2 (Cyc A positive, EdU positive) nuclei are indicated (arrows). (TIF) [file pgen.1003226.s003.tif]

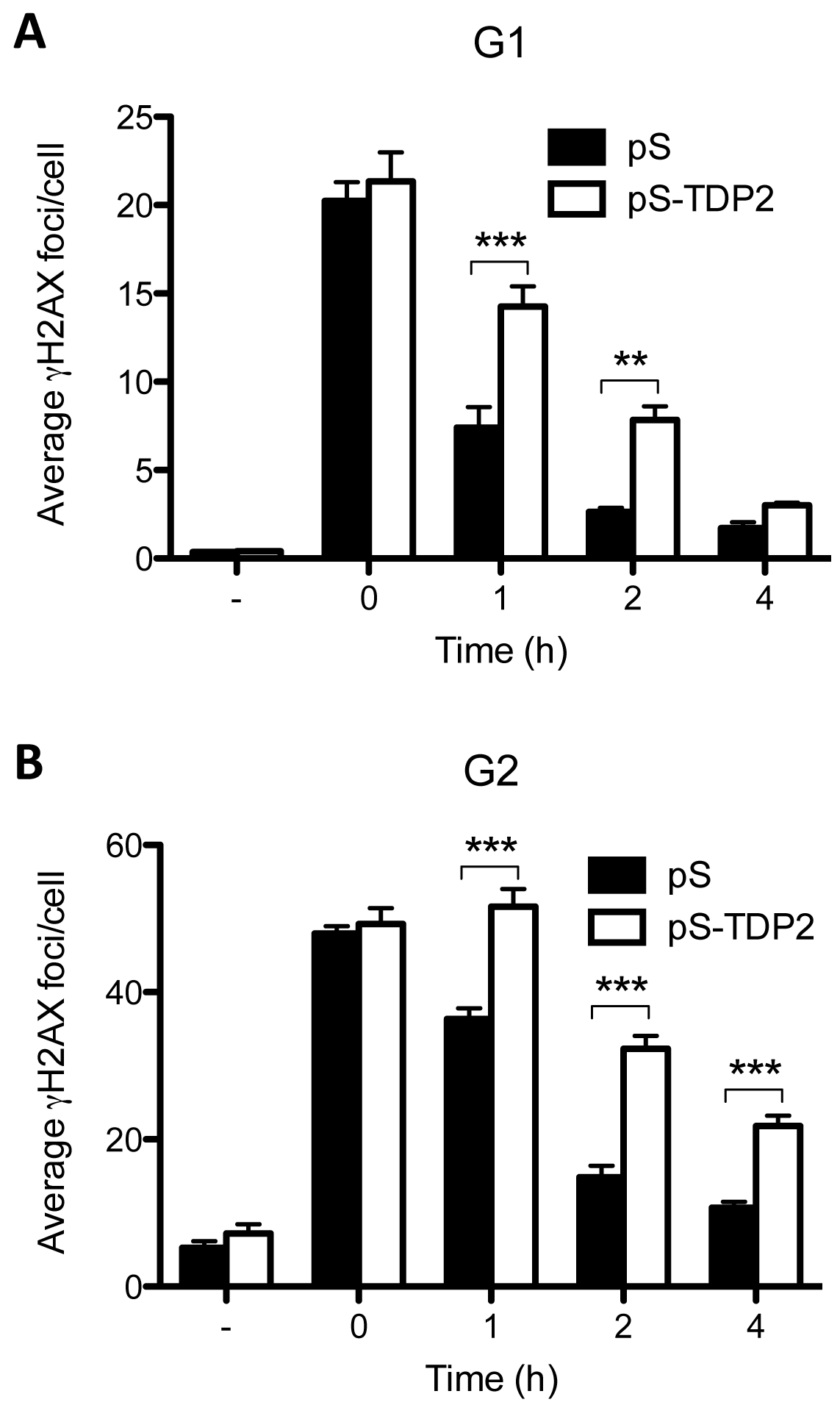

Supplement: Figure S4 — TDP2 depletion impairs repair of TOP2-induced DSBs in human A549 cells. A. γH2AX foci induction after 30 min 50 µM etoposide treatment and repair at different times following drug removal in G1 TDP2-depleted (pS-TDP2) and control non-depleted (pS) cells. B. G2 cells with 10 µM etoposide treatment. Other details as in “A”. Average ± s.e.m. of at least three independent experiments is shown. Statistical significance by Two-way ANOVA test with Bonferroni post-test is indicated. TDP2 depletion was performed as previously described [22]. (TIF) [file pgen.1003226.s004.tif]

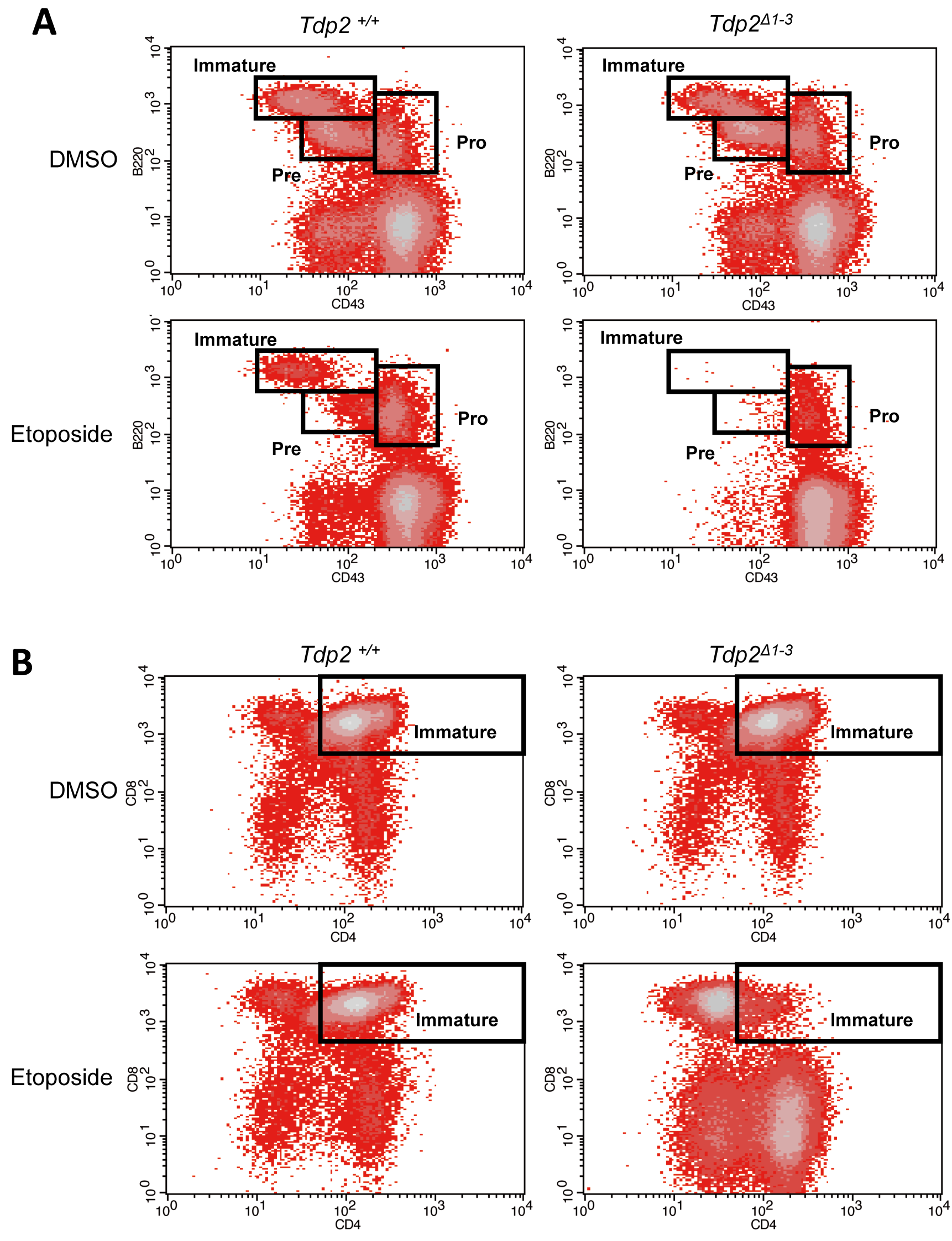

Supplement: Figure S5 — The absence of TDP2 sensitizes lymphocyte precursors to etoposide treatment in vivo. FACS analysis of B-cells in bone marrow (A) and T-cells in thymus (B) in wild-type and Tdp2Δ1–3 animals 6 days after treatment with 75 mg/kg etoposide or vehicle control (DMSO). Pro-B cell (CD43+ B220+), Pre-B cell (CD43− B220low) immature B cell (CD43− B220high) and immature T cell (CD4+ CD8+) populations are indicated (rectangles). (TIF) [file pgen.1003226.s005.tif]

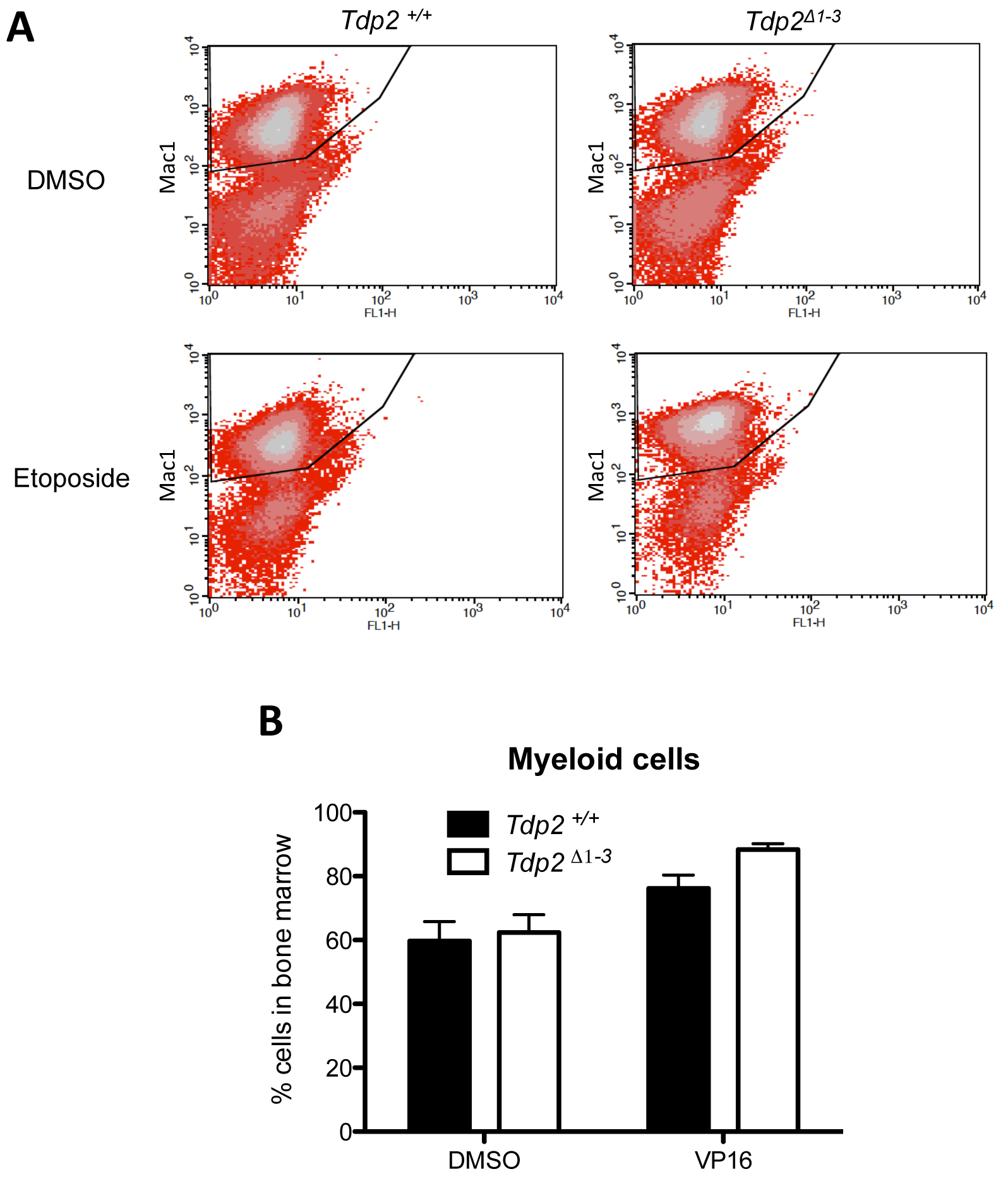

Supplement: Figure S6 — Myeloid cells are not significantly affected by etoposide treatment. FACS analysis of Mac1+ myeloid cells in bone marrow in wild-type and Tdp2Δ1–3 animals 6 days after treatment with 75 mg/kg etoposide or vehicle control (DMSO). Scatter plot (A) and quantification (B) are shown. The mild increase correlates with the observed decrease in lymphocite precursors. (TIF) [file pgen.1003226.s006.tif]
